# Noise-induced hearing loss vulnerability in type III intermediate filament peripherin gene knockout mice

**DOI:** 10.3389/fneur.2022.962227

**Published:** 2022-09-26

**Authors:** Jennie M. E. Cederholm, Kristina E. Parley, Chamini J. Perera, Georg von Jonquieres, Jeremy L. Pinyon, Jean-Pierre Julien, David K. Ryugo, Allen F. Ryan, Gary D. Housley

**Affiliations:** ^1^Translational Neuroscience Facility and Department of Physiology, School of Biomedical Sciences, UNSW Sydney, Sydney, NSW, Australia; ^2^Department of Psychiatry and Neuroscience, CERVO Brain Research Centre, Laval University, Quebec, QC, Canada; ^3^Garvan Institute of Medical Research, Sydney, NSW, Australia; ^4^School of Biomedical Sciences, UNSW Sydney, Sydney, NSW, Australia; ^5^Department of Otolaryngology, Head, Neck & Skull Base Surgery, St Vincent's Hospital, Sydney, NSW, Australia; ^6^Departments of Surgery and Neurosciences, University of California, San Diego, La Jolla, CA, United States; ^7^Veterans Administration Medical Center, La Jolla, CA, United States

**Keywords:** cochlea, contralateral suppression, type II spiral ganglion neurons, medial olivocochlear (MOC) efferents, distortion product otoacoustic emission (DPOAE)

## Abstract

In the post-natal mouse cochlea, type II spiral ganglion neurons (SGNs) innervating the electromotile outer hair cells (OHCs) of the ‘cochlear amplifier' selectively express the type III intermediate filament peripherin gene (*Prph)*. Immunolabeling showed that *Prph* knockout (KO) mice exhibited disruption of this (outer spiral bundle) afferent innervation, while the radial fiber (type I SGN) innervation of the inner hair cells (~95% of the SGN population) was retained. Functionality of the medial olivocochlear (MOC) efferent innervation of the OHCs was confirmed in the *Prph*KO, based on suppression of distortion product otoacoustic emissions (DPOAEs) *via* direct electrical stimulation. However, “contralateral suppression” of the MOC reflex neural circuit, evident as a rapid reduction in cubic DPOAE when noise is presented to the opposite ear in wildtype mice, was substantially disrupted in the *Prph*KO. Auditory brainstem response (ABR) measurements demonstrated that hearing sensitivity (thresholds and growth-functions) were indistinguishable between wildtype and *Prph*KO mice. Despite this comparability in sound transduction and strength of the afferent signal to the central auditory pathways, high-intensity, broadband noise exposure (108 dB SPL, 1 h) produced permanent high frequency hearing loss (24–32 kHz) in *Prph*KO mice but not the wildtype mice, consistent with the attenuated contralateral suppression of the *Prph*KO. These data support the postulate that auditory neurons expressing *Prph* contribute to the sensory arm of the otoprotective MOC feedback circuit.

## Introduction

Mammalian sound perception requires mechanoelectrical transduction by cochlear inner hair cells (IHCs), each discretely innervated by multiple type I spiral ganglion afferent neurons (type I SGN). These afferents represent ~95% of the cochlear primary afferents synapsing in the cochlear nucleus region of the brainstem, where their synchronous firing in response to sound stimuli is evident as waves I and II of the evoked potential auditory brainstem response (ABR). The adjacent outer hair cells (OHCs) lend critical hearing sensitivity (~40 dB, or 100-fold) (1), converting sound-evoked receptor potentials into electromechanical force, amplifying and shaping basilar membrane vibration as a “cochlear amplifier” (2). OHC–specific electromotility can be directly measured as otoacoustic emissions using a sensitive microphone placed in the ear canal. The cochlear amplifier is subject to dynamic efferent neural feedback, which contributes broadly to hearing performance, including attention to sound, sound localization, hearing perception plasticity, improved hearing discrimination in noise, and protection of hair cells and their auditory synapses from acoustic overstimulation (otoprotection) (3, 4). The principal neural feedback circuit to the OHCs involves the contralateral medial olivocochlear (MOC) efferent neurons located within the superior olivary complex - ventral nucleus of the trapezoid body in the brainstem. MOC axons form the crossed olivocochlear bundle (COCB), which passes across the floor of the fourth ventricle, projecting to the ipsilateral cochlea, to synapse with the OHCs (5, 6). Through this MOC projection, contralateral acoustic stimulation strongly inhibits the ipsilateral cochlear amplifier (‘contralateral suppression') (4, 7–9). It is generally thought that the sensory input from the contralateral cochlea driving the MOC feedback circuit arises from the IHC - type I SGN afferents (10, 11). This position is primarily founded on studies in guinea pig and cat where single MOC efferent fibers show recruitment from low sound levels (20 dB SPL), with sharp tuning curves and characteristic frequencies similar to those of adjacent type I SGN afferents (12, 13). However, evidence is emerging that at high sound levels, where MOC efferent feedback confers protection from noise-induced hearing loss (14), input from the OHC–type II SGN pathway may complement the IHC - type I SGN drive of the MOC efferent feedback circuit. For example, contralateral suppression was maintained following ouabain treatment of the cochlea that selectively ablated type I SGN (reducing ABR amplitude), while leaving the type II SGN innervation of OHCs intact (15). Further, type II SGN drive to the cochlear nucleus region known to connect with the MOC efferent arm of the circuit noise has recently been established for noise levels relevant to MOC efferent - mediated otoprotection (16).

The type II SGN are a small, enigmatic subpopulation (~ 5%) of unmyelinated afferents that exclusively innervate the OHCs (17, 18). Their peripheral neurites project radially past the IHCs to form outer spiral bundles (OSB) that run basally, each branching to provide *en passant*, afferent synapses on multiple OHCs. This distributed receptive field overlaps with the frequency map of the cochlear amplifier, which spreads for ~¼ octave toward the basal (high frequency) region, corresponding to the active region of the cochlear amplifier (6, 19, 20). This is also congruent to a basal shift in the MOC efferent activation, which overlaps with the cochlear amplifier region as well as the type II SGN afferent map as sound levels increase (6, 21). The correspondence between the distribution of type II SGN afferent terminals and OHC-dependent active biomechanics prompted Kim (22) to propose that type II SGN contribute to the afferent input for the MOC efferent innervation of the OHCs.

In a prior study we investigated the afferent arm of the MOC feedback neural circuit using a peripherin knockout (*Prph*KO) mouse model (23). Peripherin is a type III intermediate filament protein associated with sensory fiber development, and in the mouse cochlea, peripherin expression is limited to the type II SGN post-natally (24, 25). The Froud et al. (23) study demonstrated disruption of the type II SGN innervation of the OHCs and an associated attenuation of contralateral suppression. A subsequent examination of the *Prph*KO mouse model challenged the morphological phenotype and postulated that the reported disruption of the MOC reflex may have been due to selective effects of loss of *Prph* expression by MOC neurons (11). In the present study we show that the IHC - type I SGN afferents and MOC efferent fibers remain viable in the *Prph*KO mouse model, while the disruption of the type II SGN afferent innervation of the OHC in *Prph*KO mice is more profound than indicated in our initial report (23). This selective disruption of the afferents innervating the cochlear amplifier is associated reduction in the MOC efferent reflex and loss of otoprotection from acoustic overstimulation.

## Materials and methods

### Animals

Male and female adult 129Sv/C57BL/6 wildtype (WT) and *Prph*-null (*Prph*KO) mice on the same background were used for this study. For acoustically–evoked contralateral suppression hearing studies, and noise-induced hearing loss studies, mice were anesthetized using intraperitoneal injections (i.p.) of a ketamine (40 mg/kg)/xylazine (8 mg/kg)/acepromazine (0.5 mg/kg) (k/x/a) cocktail. Further k/x/a was administered (half the initial k/x/a dose mixed with the equivalent volume of 0.9% saline) as required to keep the mice anesthetized throughout the experiment (typically every 30 min). For the electrically-evoked MOC stimulation experiments mice were anesthetized with an initial k/x/a injection as above, then kept anesthetized with 0.5–1% isoflurane supplemented with O_2_ throughout the experiment. The level of anesthesia and the O_2_ saturation of the mice were monitored using a pulse oximetry system (MouseOx, STARR Life Sciences, SDR Scientific, Australia). Mice were kept on a heat pad for the duration of the experiment with their core body temperature clamped at 37°C by feedback control (Right Temp, Able Scientific, Perth, Australia). Ophthalmic ointment was applied to the eyes once the animal was anesthetized to prevent corneal drying. At the completion of the studies, the animals were euthanised using pentobarbital (Virbac Australia, 100 mg/kg of body weight at 100 mg/ml, i.p.). Experiments were conducted according to UNSW Sydney Animal Care and Ethics Committee approved protocols, which conform to the Australian code for the care and use of animals for scientific purposes (26). The design and communication of the study align with the ARRIVE guidelines (27).

### Genotyping

The *Prph*KO mouse model was established in 2001 using 129Sv strain embryonic stem cells bearing the peripherin knockout construct (exon 1 deletion) that were injected into C57BL/6 strain blastocysts (28). To produce heterozygous *Prph*KO mice, the generated chimeric mice were crossed with C57BL/6 mice. These heterozygous mice were used as breeders to provide littermates of identical genetic background. The *Prph*KO mouse line was established at UNSW in 2008. PCR-based genotyping utilized the following primer sets: Prph-5′-UTR-F: 5′ GCT ATA AAG CCG CCC CGC ATC 3′; Prph-exon1-R: 5′ AGG GCT GCG TTC TGC TGC TC 3′; LacZ-R: 5′ GTC CTG GCC GTA ACC GAC CC 3′; Imaging of the PCR amplicons following agarose gel electrophoresis was used to distinguish WT (452 bp), KO (640 bp) and heterozygous (452 + 640 bp bands) mice (Supplementary material 1A). Homozygous knockout genotype validation was confirmed by the absence of peripherin immunolabelled cochlear type II SGN [after (23)] (Supplementary material 1B,C).

### Immunohistochemistry

Mouse cochleae were dissected and fixed by scali perfusion of 4% paraformaldehyde, decalcified for 14 days in 8% EDTA and then cryoprotected using 30% sucrose. The cochleae were mounted (Optimum Cutting Temperature (O.C.T) compound, Tissue-Tek, Sakura Finetek, Torrance, CA, USA) and cryosectioned at 50 μm. For wholemount preparations, cochleae were dissected into 2–4 pieces at the apical and basal turns. In both free-floating cryosections and the wholemounts, non-specific binding was blocked with 10–15% normal goat or donkey serum, 1 % Triton X−100 in phosphate buffered saline (PBS) for 1 h at room temperature (RT). Sections were then immersed in primary antibodies: Neurofilament heavy polypeptide (NF200, Sigma, Cat# N4142, RRID: AB_477272; rabbit, 1:5000); C-terminal-binding protein 2/RIBEYE ribbon synapse maker (29) (CtBP2, BD Bioscience, Cat# 612044, RRID: AB_399431; mouse, 1:500); Tubulin beta-3 chain (β-III tubulin) (TuJ1, Covance, Cat# MMS-435P, RRID: AB_2313773; mouse, 1:1000); Peripherin (Everest Biotech, Cat# EB12405, RRID: AB_2783842; goat, 1:1000); Parvalbumin alpha (Swant, Cat# PVG-213, RRID: AB_2650496; goat, 1:1000); Vesicular acetylcholine transporter (VAChT) (Phoenix Pharmaceuticals Inc., Cat# H-V007, RRID: AB_2315530; rabbit, 1:100); Myosin 7A (Proteus, Cat# 25-6790, RRID: AB_10015251; rabbit, 1:500); AMPA subtype 2 glutamate receptor (GluA2, Millipore, Cat# MAB397, RRID: AB_2113875; mouse, 1:1000) in 5–10% normal goat or donkey serum, 0.1% Triton X - 100 in PBS, overnight at RT. Sections were then washed in PBS and appropriate secondary antibody was applied overnight at RT [anti-rabbit IgG AlexaFluor 594, anti-mouse IgG AlexaFluor 488/594/647 (1:1000) or anti-goat IgG AlexaFluor 488 (1:1000) (Molecular Probes)], 5% normal goat or donkey serum in PBS. Following a further PBS wash, nuclear labeling was achieved by incubating the sections for 5 min in DAPI (4′,6-diamidino-2-phenylindole; 1:5000; Sigma). Two final washes in PBS were performed before the sections were mounted on glass slides using Vectashield (Vector Laboratories) and imaged on a Zeiss confocal microscope (Zeiss 710 NLO). Data were obtained from 1–3 organ of Corti z stacks (30 μm) analyzed per animal, typically 3 animals per group. Data for the quantitative analysis of ribbon synapses was obtained using 30 μm stacks of confocal images; 63x oil immersion objective; mid-cochlear level, parsing the position of the CtBP2-labelled puncta within 2.5 μm bins relative to the equator line of the DAPI-labelled OHC nuclei. These data were blinded, randomized, and double scored before decoding.

### Hearing function tests

Hearing testing was carried out in a sound-attenuating chamber (Sonora Technology, Japan) using an auditory-evoked potential and DPOAE workstation (TDT system 3 with RX6 and RX6-2 signal processors, Tucker Davis Technologies, Ft Lauderdale, FL, USA) with BioSig32 software. Sound levels were calibrated using a one-quarter-inch Free Field Measure Calibration Microphone (model 7016; ACO Co Ltd., Japan).

#### Acoustically-evoked contralateral suppression

The contralateral ear was exposed to broadband suppressor sound stimulation (96 dB SPL noise, 15–25 kHz, 15 s; *n* = 7 each for WT and *Prph*KO; 82 dB SPL noise, 10–17 kHz, 60 s; *n* = 8 each for WT and *Prph*KO) using a MF1 speaker (TDT), while cubic (2*f*_1_-*f*_2_) DPOAEs were detected using a microphone coupled to the ipsilateral ear canal (ER-B10+, Etymotic Research, IL, USA), alongside two EC1 electrostatic speakers (TDT), controlled by the TDT system 3 workstation. DPOAEs were elicited using equal primary tones (*f*_1_ and *f*_2_; *f*_2_/*f*_1_ ratio: 1.25) around 20 kHz at 60 dB (for the 96 dB SPL noise study) or around 28 kHz at 65 dB (for the 82 dB SPL noise study). In total 10–50 measurements were averaged (6.7/s) for each recording. DPOAE measurements were taken before (baseline), during and following (recovery) suppressor stimulus.

#### Electrically-evoked contralateral suppression

To directly drive the contralateral MOC efferent innervation of the OHCs, these fibers were electrically stimulated at the point where they cross the midline on the floor of the fourth ventricle of the brainstem. Mice were initially anesthetized with a k/x/a cocktail, maintained on a heat pad and eye ointment applied as described in the section *Animals*. This was followed by shaving the head to provide a clean surgical field for the dorsal-occipital approach to the fourth ventricle. A tracheostomy was performed, and the animals were ventilated throughout the experiment with a respiration rate of 100 breaths per minute and 10 cm peak inspiratory pressure (Kent Scientific TOPO™ Dual Mode Ventilator, Torrington, CT, USA). The animal was then positioned onto a teeth bar to stabilize the head position. The dorsal brainstem was accessed by removing muscles and connective tissue between the C1 (Atlas) vertebra and the occiput. This was followed by an incision in the dura covering the foramen, and the stimulating electrodes (two platinum iridium wires with 500 μm exposed tips, 400 μm separation) were positioned with one electrode on the midline of the fourth ventricle floor and the second electrode ipsilateral, using a micromanipulator. Twitching of the ears in response to a test electrical stimulation (monophasic 150 μs pulses, 200 Hz) ~ 5s, indicated the correct position. α-D-tubocurarine (1.25 mg/kg, i.p.) was then administered to achieve muscle paralysis, which included paralysis of the stapedius reflex. The right (ipsilateral) external auditory meatus of the mouse was coupled to the DPOAE probe. A baseline measurement was obtained as the cubic DPOAE around 16 kHz at 50 or 55 dB SPL primaries (*f*_1_ = *f*_2_ amplitude, producing DPOAEs ~ 10 dB above the noise floor); 20 averaged measurements (10 samples each) over 1 min. The electrically-evoked contralateral suppression was then recorded during 25 seconds of stimulation (8 data point averages), followed by 2.5 min of recorded recovery. The DPOAE amplitudes were analyzed relative to the noise floor. These experiments proved particularly challenging, with 3/16 WT mice and 3/7 *Prph*KO mice providing useable data. However, electrically-evoked DPOAE suppression for each of the successful WT and *Prph*KO mice (*n* = 3 mice per genotype) were obtained from 3–5 repeats per mouse.

#### Comparison of vulnerability to noise-induced hearing loss

To assess the otoprotection conferred by OHC–type II SGN–mediated MOC efferent cochlear amplifier suppression, noise-induced hearing loss was assessed in *Prph*KO vs. WT mice using auditory brainstem responses (ABRs) and cubic DPOAE measurements with acute noise presentation. For ABR, following k/x/a anesthesia induction, subdermal platinum electrodes were inserted subcutaneously at the vertex (+), over the mastoid process (–), and in the hind flank (ground) [after (30)]. Click (100 μs alternating polarity) or pure tone pips (4, 8, 16, 24, and 32 kHz) stimuli (5 ms, 0.5 ms rise/fall time, 10/s) were delivered using an EC1 electrostatic speaker, with the generated ABR potentials amplified, filtered and averaged 512 times using the TDT System 3 workstation. The ABR threshold for each frequency was determined as the lowest intensity (5 dB steps from 70 dB SPL) at which the P2 ABR wave could still be visually observed above the noise floor (~100 nV). Cochleae were then exposed to 108 dB SPL “open field” broadband white noise (4–32 kHz; 2^nd^ order Bessel filter) for 1 h. The white noise stimulus was generated using custom software with a National Instruments A/D driving an amplifier (BIEMA model Q250, Altronic, Northbridge, WA, Australia) and delivered *via* an MF1 speaker (TDT) positioned at midline, 15 cm in front of the mouse (*n* = 9 for each genotype). The noise was calibrated at ear level. ABR threshold shifts were determined immediately post-noise by remeasurement before the mice recovered from the anesthesia. The mice were then rested for 14 days before being re-tested to determine sustained hearing loss [permanent threshold shift (PTS), after (31)].

### Data analysis

Data are presented as the population mean ± s.e.m.. Statistical analysis was performed using a one-way Analysis of Variance (ANOVA), a two-way repeated measures (RM) ANOVA (Sigmaplot^®^, Systat Software Inc., San Jose, CA, USA), a two-way ANOVA or *t*-test, as indicated; significance at alpha ≤ 0.05. Data were tested for normal distribution and Holm-Sidak *post hoc* analysis was utilized for multiple pairwise comparisons within ANOVA. Grubbs' test was used to assess data outliers (GraphPad software, San Diego, CA, USA). Data were graphically presented using Sigmaplot^®^ software.

## Results

### Disruption of the outer spiral bundle (type II SGN) fiber tracts in *Prph* knockout cochleae

**Figure 1:**
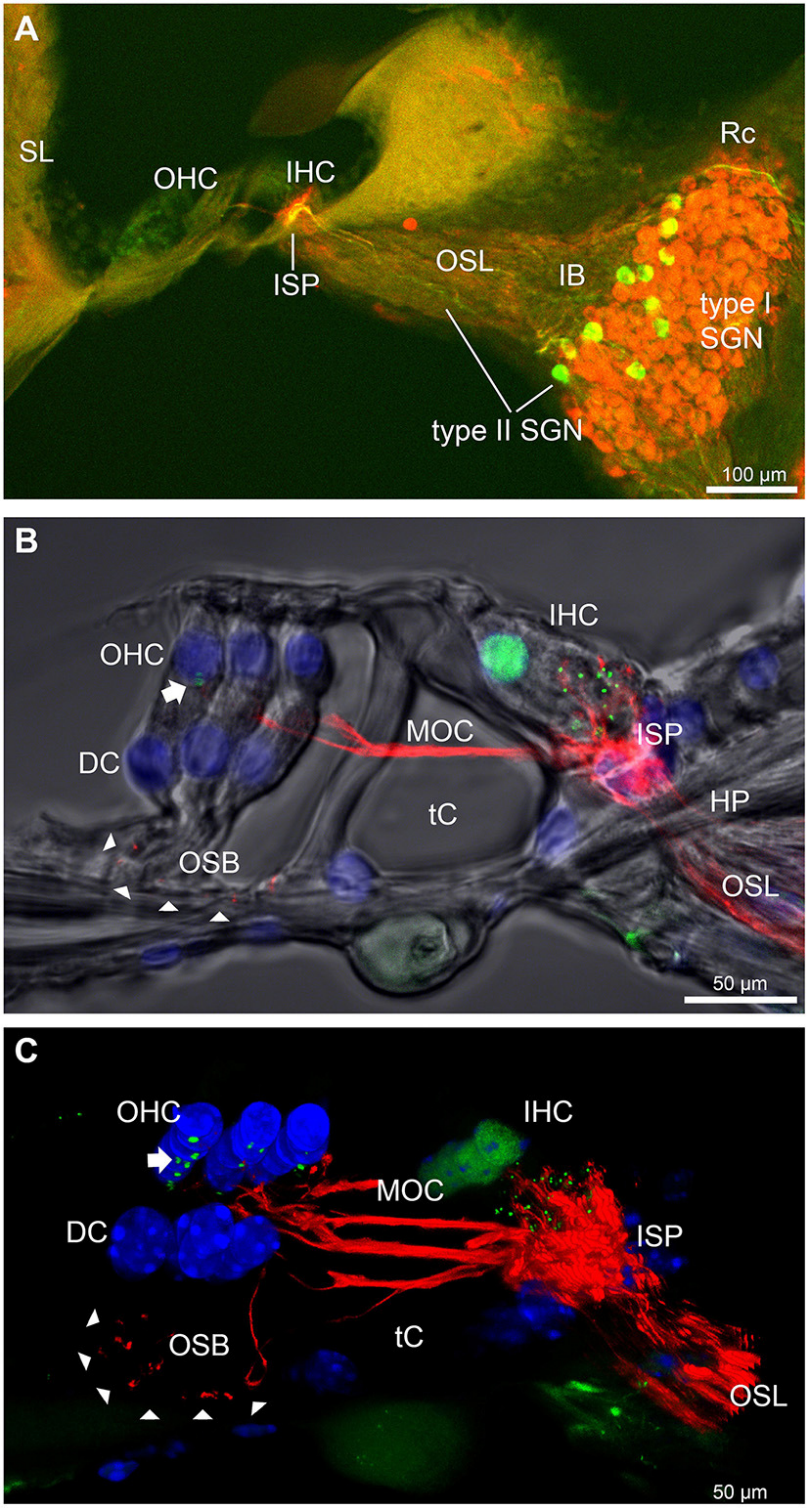
Immunofluorescence labeling of the afferent and efferent innervation of the organ of Corti in the wildtype mouse cochlea. **(A)** Spiral ganglion neuron (SGN) somata and neurites (type I SGN (red) β-III tubulin immunofluorescence; type II SGN (green/yellow) peripherin immunofluorescence). The type II SGN sub-population is biased to the lateral aspect of Rosenthal's canal (Rc), proximate to the intraganglionic spiral bundle (IB); the IB contains the medial olivocochlear (MOC) efferent axons from the superior olivary complex of the brainstem. All three nerve fiber types project to the organ of Corti *via* the osseous spiral lamina (OSL). The inner spiral plexus (ISP) is located at the basal pole region of the inner hair cells (IHC) and predominantly reflects type I SGN terminals. OHC, outer hair cells; SL, spiral ligament. **(B)** Detail of the innervation and pre-synaptic ribbon complexes of the hair cells within the organ of Corti, delineated using NF200 (red) immunofluorescence for the nerve fibers and CtBP2/RIBEYE (green) immunofluorescence for the ribbons. Single confocal optical section overlaid with transmitted light image. NF200 immunolabelling delineates type II SGN neurites as discrete outer spiral bundle (OSB) fiber tracts (delineated by arrowheads) beneath the OHCs and their supporting Deiters' cells (DC). Nuclei labelled with DAPI (blue). MOC efferent fibers cross the tunnel of the organ of Corti (tC) to innervate the OHCs. Note synaptic ribbons basal to the OHC nuclei (arrow). In comparison, the sub-nuclear domain of the IHCs contains many more CtBP2 immunopositive synaptic ribbons, each of which is aligned to a single type I SGN neurite terminal. Habenula perforata (HP). **(C)**, Confocal immunofluorescence reconstruction of a 50 μm cryosection delineates cell structure within the organ of Corti. The CtBP2 immunopositive synaptic ribbons are localized in a highly regular pattern beneath the mid-ventral aspect of the OHC nuclei (one or two per OHC; arrow), whereas each IHC contains > 10 CtBP2 puncta. The type II SGN outer spiral fibers within the OSB are clearly delineated (arrowheads) by the NF200 immunofluorescence ventral to the DCs. See also Supplementary material 2 for 3D rendering.

**Figure 2:**
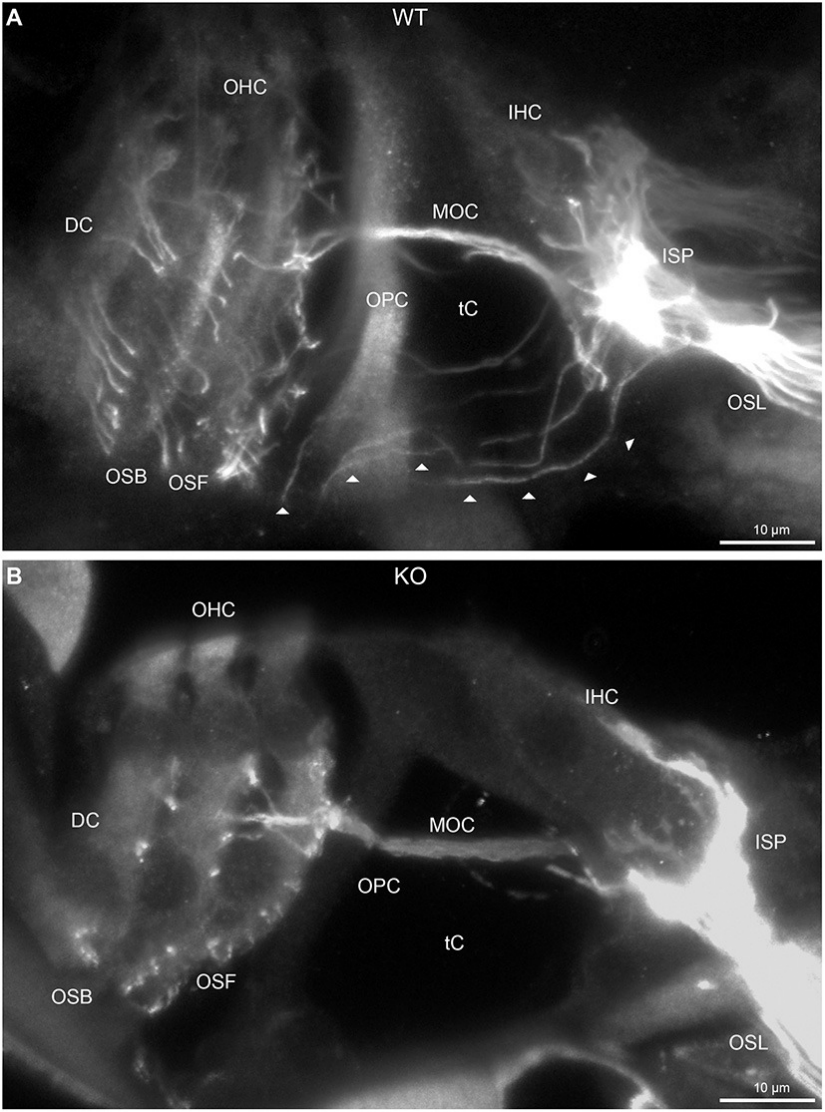
Disruption of type II spiral ganglion afferents, the outer spiral fibers (OSF), in the mid-basal region of the *Prph* knockout (KO) delineated using β-III tubulin immunofluorescence. **(A)** In the WT cochlea, the type II spiral ganglion afferents exit from the basal aspect of the inner spiral plexus (ISP) beneath the inner hair cells (IHC), cross the floor of the tunnel of Corti (tC) (arrowheads), pass between the outer pillar cells (OPC) and turn basally to form parallel sheets of OSF along the sides of the Deiters' cells (DC), as the outer spiral bundles (OSB). The OSF branch periodically and turn apically to terminate along the three rows of outer hair cells (OHC). The medial olivocochlear (MOC) efferent fibers cross the mid-region of the tC to innervate the OHC. **(B)** In the *Prph*KO image, the OSF density is substantially reduced, while equivalent MOC efferent fiber projections extend to the OHC. OSL, osseous spiral lamina. Transparent-mode confocal reconstructed images of batch-processed WT and *Prph*KO cryosections; 21–25 μm depth z stacks. See also Supplementary material 2.

**Figure 3:**
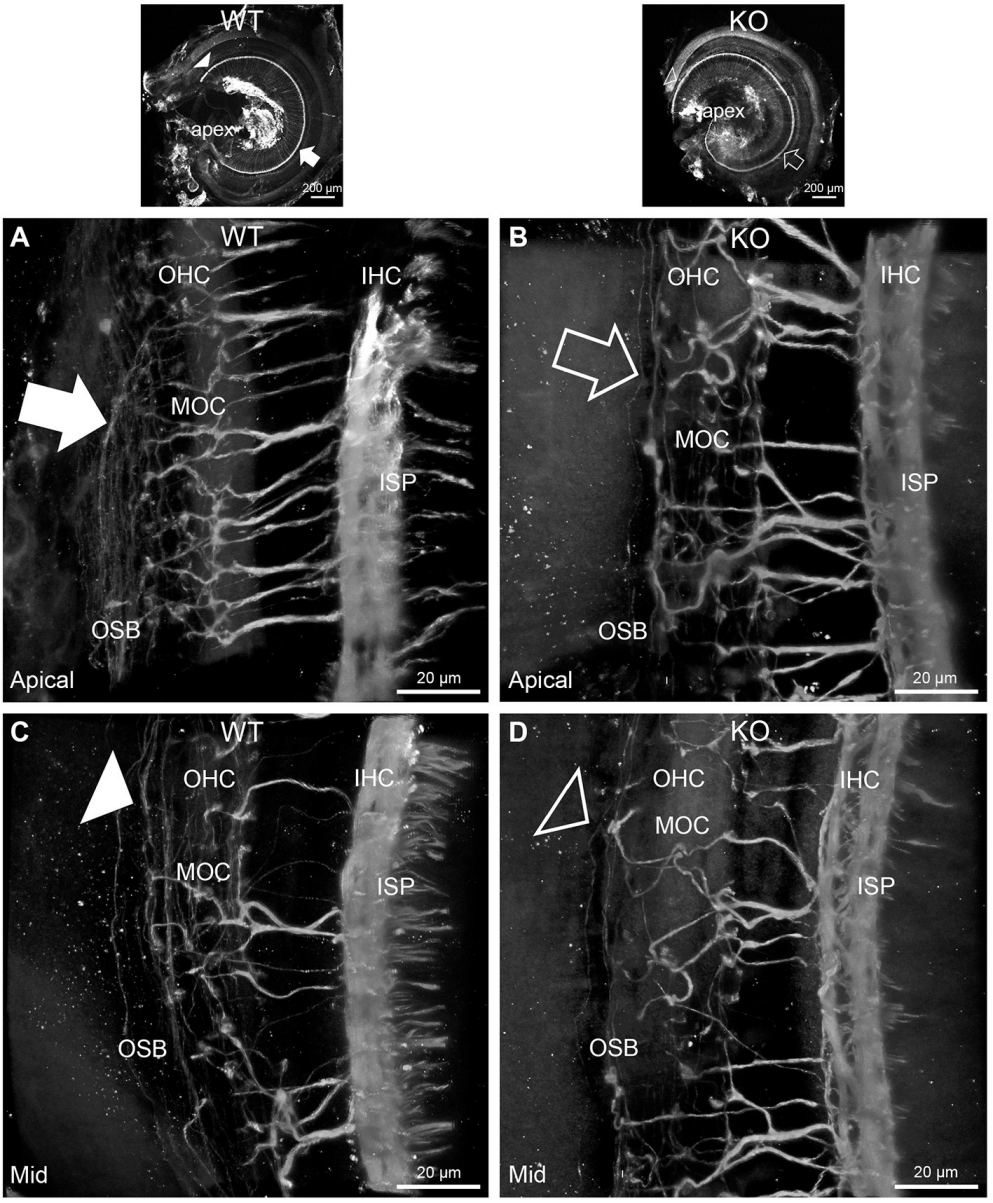
Disruption of the outer spiral bundle structure (OSB; type II spiral ganglion neurites form the outer spiral bundles) in *Prph* knockout (KO) vs. wildtype (WT) mouse cochleae from apex to mid region. Arrows in the low-power images indicate the corresponding regions of these whole-mount preparations which are shown at high resolution. The outer spiral fibers run parallel to the tunnel of Corti within the OSB (arrows), whereas the MOC fibers project across the tunnel from the inner spiral plexus (ISP) region to the outer hair cell (OHC) region, where they branch to innervate multiple OHCs. Note the comparatively higher number of outer spiral fibers in the apical region in the WT **(A)** (filled arrow), compared with the mid-cochlear region in the same tissue **(C)**, **(**filled arrowhead). The corresponding regions in the wholemount KO cochlea **(B,D);** (open arrow and open arrowhead) show minimal labeling in the OSB. Note the equivalency between KO and WT, of the medial olivocochlear (MOC) efferent axon projections to the OHCs. Maximum intensity projection β-III tubulin immunofluorescence confocal images of the organ of Corti (imaged from the basilar membrane surface to optimally resolve the OSB). IHC, inner hair cell region. See also Supplementary materials 4, 5 for 3D rendering.

**Figure 4:**
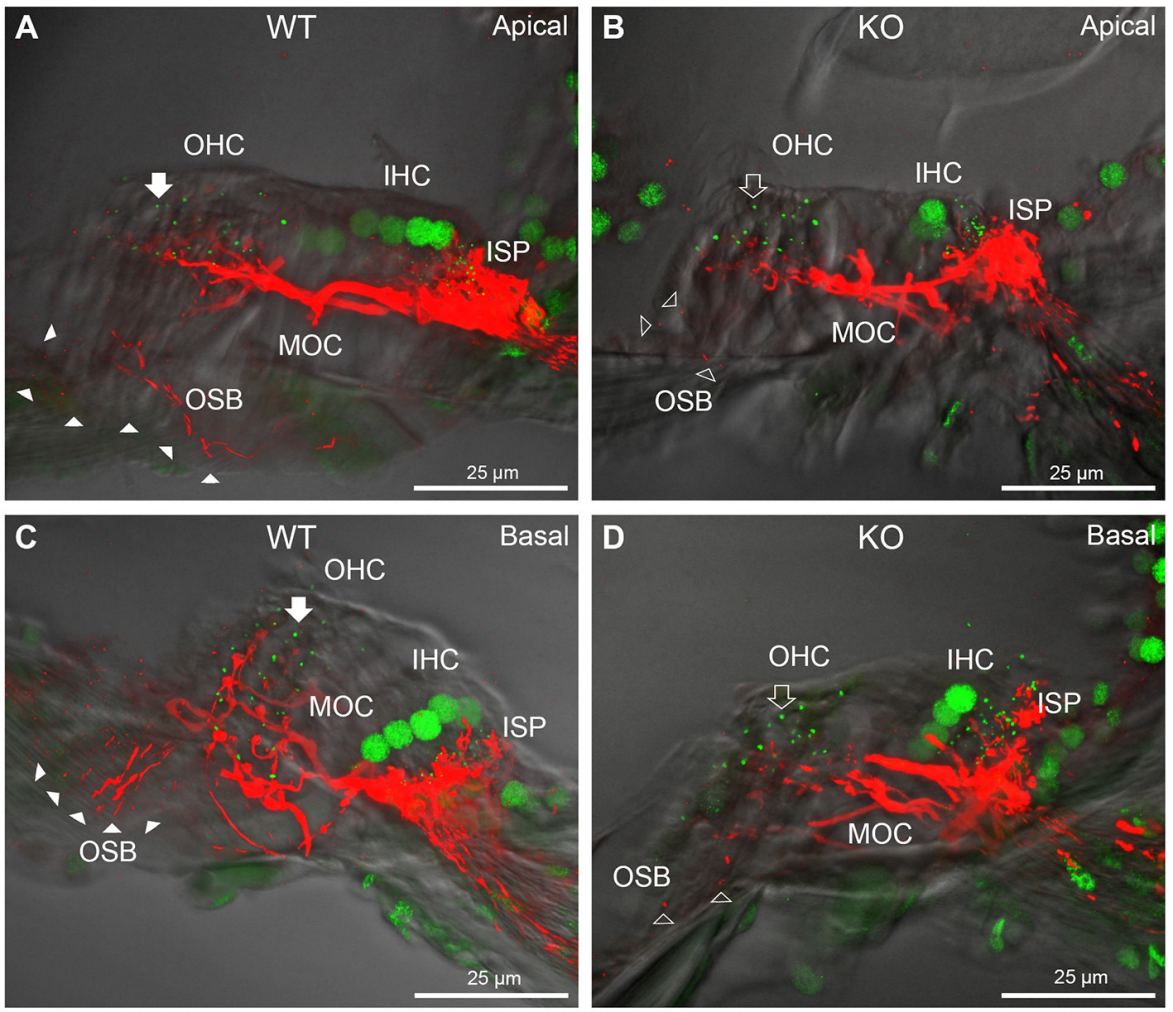
Comparison of NF200 (red) immunolabelling of outer spiral bundle (OSB) structure of the type II SGN neurites projecting to the outer hair cells (OHC) from wildtype (WT, filled arrow heads) and *Prph*KO (open arrow heads) cochleae. Note the limited OSB representation in the KO tissue from all regions. Images from single mid-modiolar cryosections for each of WT and KO illustrate the OSB fiber density within the organ of Corti at the apex **(A,B)** and base **(C,D)**. CtBP2/RIBEYE immunofluorescence (green puncta) delineates the pre-synaptic ribbons in both the OHCs and inner hair cells (IHC). The regular pattern of pre-synaptic ribbons at the mid-basal region of the three rows of OHCs evident in the WT (filled arrow), is disrupted in the KO (open arrow). The medial olivocochlear (MOC) efferent fiber innervation of the OHCs lies above the OSB. The dense type I SGN fiber synaptic complex at the base of the IHC (inner spiral plexus, ISP) is juxtaposed to the high density of synaptic ribbons in both WT and KO tissue. In total 50 μm cryosections; confocal projection images. See also Supplementary material 6.

The type II SGN somata sub-population was specifically identified using peripherin immunofluorescence. The neurons were predominantly located in the lateral aspect of Rosenthal's canal, juxtaposed to the intraganglionic spiral bundle of efferent fibers (*n* = 6 WT). As previously reported (23), the anti-peripherin fluorescence signal declined with age, particularly in the peripheral neurites (see adult WT cochlea Figure 1A; and post-natal day 8 cochlea (*n* = 3 WT), Supplementary material 1C). β-III tubulin and NF200 immunolabelling was employed for comparison of the representation of type I and type II SGN neurite projections within the adult mouse cochlea across wildtype (WT) and *Prph*KO tissue (Figures 1A–C; Supplementary material 2. The type II neurites track alongside the type I radial fibers within the osseous spiral lamina. Both types of afferent neurites pass through the habenula perforata and project toward the base of the inner hair cells (inner spiral plexus, ISP). The β-III tubulin and NF200 immunofluorescence labelled type II SGN neurites extended beyond the inner hair cells, with the fibers crossing the tunnel of Corti well beneath the extensive medial olivocochlear (efferent) bundle fibers (MOC) (Figures 1–4; Supplementary material 3). The type II fibers then became basally projecting outer spiral fibers within the OSB beneath each of the three rows of OHCs and their associated Deiters' cells (DC). The outer spiral fibers periodically separate from the OSB, to ascend between the DC processes and make afferent synapses at the bases of OHCs (for example, Figures 1B,C, 2A).

In *Prph*KO cochleae, the immunolabelling of the type II SGN afferent innervation was distinctly disrupted, whereas the type I SGN innervation and the MOC efferent fiber representation were normal in appearance. Comparisons between *PrphKO* tissue and corresponding WT tissue utilized batch immunoprocessing and imaging (Figures 2–4; Supplementary material 3; Supplementary material 4; Supplementary material 5). In *Prph*KO, both β-III tubulin (Figure 2A for WT compared with Figure 2B for *Prph*KO, mid-basal region; Figures 3A,C for WT and Figures 3B,D for *Prph*KO apical and mid-regions) and NF200 (Figure 4A WT apical compared with Figure 4B *Prph*KO apical, with Figure 4C WT basal compared with Figure 4D *Prph*KO basal) labeling delineated a residual population of outer spiral fibers projecting from the ISP region, across the floor of the tunnel of Corti, and out to the OSBs in both whole-mount (*n* = 3 WT, *n* = 5 KO) and cryosectioned tissue (*n* = 6 WT, *n* = 4 KO). There was an evident gradient in this OSB pathology along the length of the organ of Corti, from an almost complete loss of these type II afferent fibers at the apex [see Figure 4B (*Prph*KO) compared with Figure 4A (WT) for apical region where OSB loss is extensive], to a partial retention of OSB fibers in the basal region [compare Figure 4D (*Prph*KO) vs. Figure 4C (WT); Supplementary material 6]. It could not be established whether this reduction in outer spiral fiber number reflected atrophy of the peripheral neurite processes of the type II SGN, or arose from loss of type II SGN cell bodies, because the peripherin immunolabelling was intrinsically absent in the *Prph*KO. However, the OSB remodeling clearly reflected disruption of the sensory neural drive from the OHCs.

**Figure 5:**
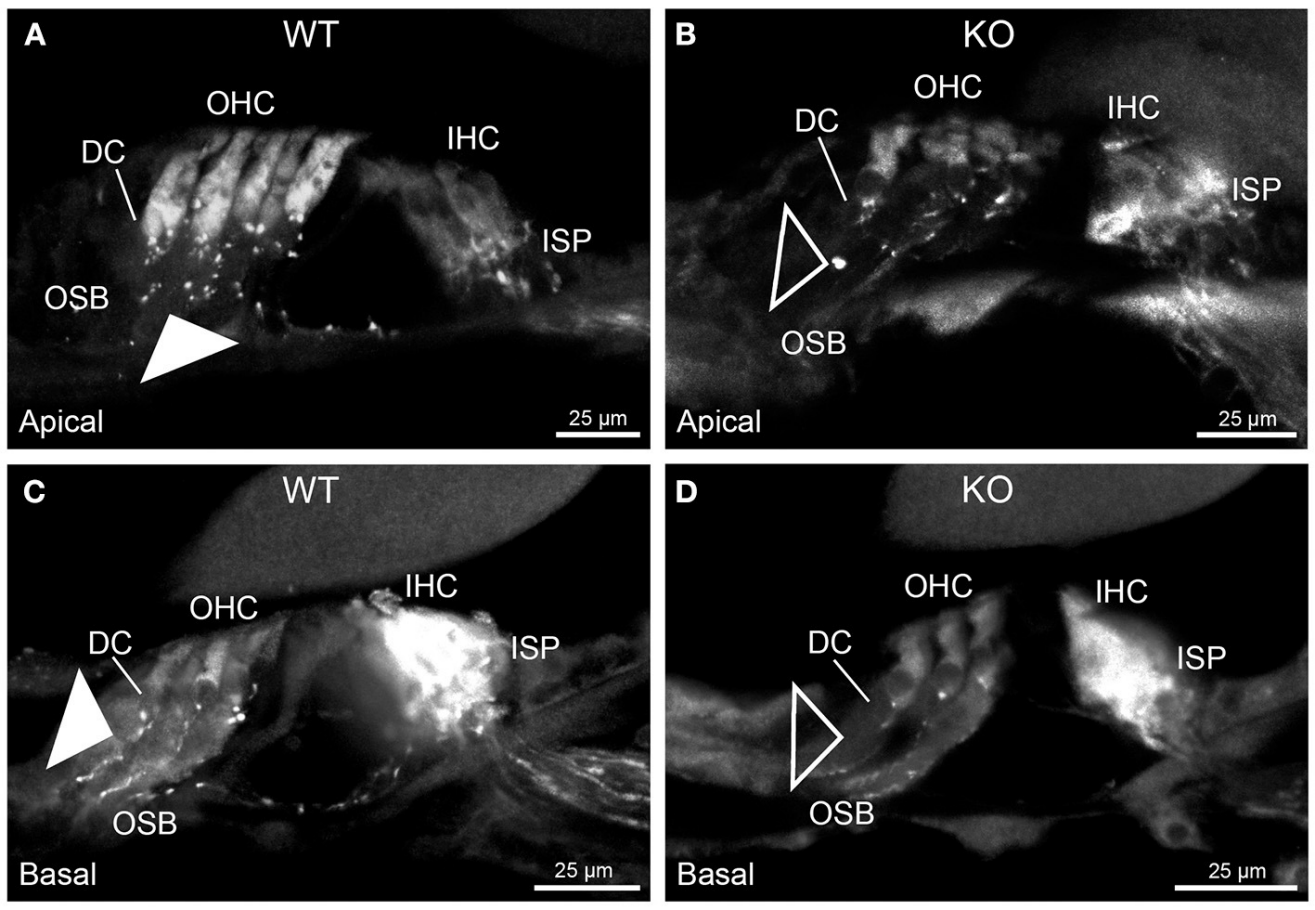
Parvalbumin immunofluorescence resolves the disruption of the type II SGN fibers (outer spiral fibers) within the outer spiral bundles (OSB) of the PrphKO mouse cochlea. Examples of batch-processed cryosections from WT **(A,C)** and KO **(B,D)** cochleae delineate the terminal regions of the outer spiral fibers, particularly with respect to their migration along the medial aspect of the Deiters' cells (DC) up to the base of the outer hair cells (OHC). Parvalbumin was also strongly labelled in the inner hair cells (IHC) in the basal region of the cochlea **(C,D)**, and in type I SGN afferent fibers within the inner spiral plexus (ISP). There was a much higher density of outer spiral fiber labeling at the level of the OSB in the WT tissue (filled arrowheads) compared with the KO tissue (open arrowheads), consistent with the β-III tubulin and NF200 immunolabelling experiments. The medial olivocochlear efferent projections to the OHCs are not delineated by this parvalbumin immunolabelling. Apical and basal regions from the same cochlea for each of WT and KO.

While clearly resolving the type II SGN afferent fibers as OSB, neither of the neuron-specific antibodies (β-III tubulin, NF200) delineated the terminal region of these fibers at the OHCs, likely reflecting limited trafficking of the β-III tubulin or the neurofilament 200 proteins to the synaptic region. Parvalbumin immunolabelling was therefore used to probe type II fiber terminations at the OHCs. Parvalbumin is a calcium binding protein that is expressed by type I and type II SGN, as well as the hair cells and supporting cells of the organ of Corti (11). This further resolved the disruption of the OHC afferent innervation, where the density of these outer spiral fiber terminal processes was substantially diminished in the *Prph*KO tissue [Figure 5A (WT apical region) compared with Figure 5B (*Prph*KO apical); Figure 5C (WT basal) compared with Figure 5D (*Prph*KO basal) (*n* = 2) *Prph*KO, batch processed with WT tissue (*n* = 3)].

### Dysregulation of the outer hair cell pre-synaptic ribbon complexes in *Prph* knockout cochleae

**Figure 6:**
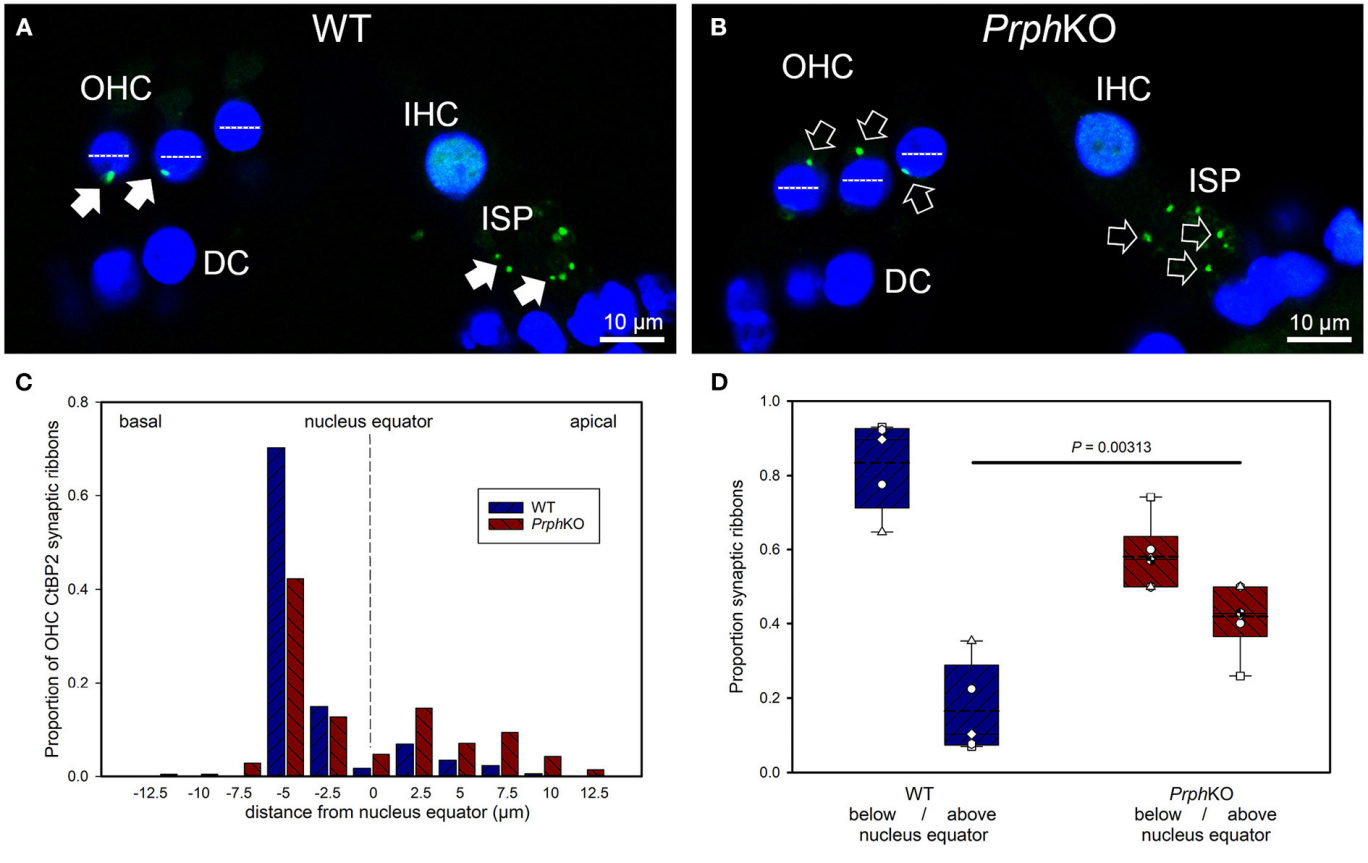
Quantitative analysis of the displacement of the outer hair cell (OHC) pre-synaptic ribbon complexes in *Prph*KO cochleae. **(A,B)** Examples of CtBP2/RIBEYE immunolabelling (green puncta, arrows) for WT and KO cochleae (50 μm cryosections; single confocal optical sections from z stacks shown). Note the apical displacement of the OHC synaptic ribbons in the KO above the equator of the nucleus (dashed lines). DC, Deiters' cells; ISP, inner spiral plexus; IHC, inner hair cell; DAPI nuclear labeling (blue). **(C)** Location of the synaptic ribbons relative to the OHC nuclei. Synaptic ribbon distribution from 175 WT OHC from 5 mice; 214 *Prph*KO OHC ribbons from 6 mice. The average number of ribbons per OHC was ~ 2. **(D)** Comparison of average distribution from the WT and KO mouse OHC ribbon complexes parsed as below or above the nucleus. The data indicate a significant migration of the synaptic ribbons away from the basal (synaptic) pole in the KO, consistent with loss of type II afferent fiber synapses (*t-*test). Box plots indicate 25 and 75% boundaries and 95% limits, with individual averages overlaid; dashed lines = means; solid lines = medians. See also Supplementary materials 7, 8.

CtBP2/RIBEYE and NF200 dual immunolabelling of *Prph*KO cochleae showed disruption of the ordered structure of the synaptic ribbon complexes at the base of the OHCs, juxtaposed to the type II SGN afferent neurite terminals. As evident in Figures 1C, 4A,C, Supplementary material 2, 1–2 CtBP2 positive puncta are normally present in the mid-basal region of each of the WT OHC, beneath the nucleus; in the *Prph*KO tissue, this regular pattern was lost (*n* = 2 WT and 2 *Prph*KO cochleae, 15–19 weeks of age; batch-processed; Figure 6A is an optical section showing the typical WT OHC synaptic ribbon location on the basolateral aspect of the OHC; Figure 6B is an example of the apical drift of these synaptic ribbons in the *Prph*KO OHCs). This abnormality was elaborated by dual immunolabelling for CtBP2 and the vesicular acetylcholine transporter (VAChT), which marks the synaptic boutons of the MOC efferent innervation of the OHCs (Supplementary material 7). In both WT (*n* = 5) and *Prph*KO (*n* = 3) mice, multiple large VAChT positive efferent boutons are present at each OHC, on the medial and mid region of the basal pole of the cells, indicating retention of the efferent MOC innervation of the OHC. In 1–3 CtBP2-labelled pre-synaptic ribbons were interposed with the MOC synapses or occupied the lateral aspect of the synaptic pole of the WT OHCs. In the *Prph*KO cochleae, the apical displacement of many of the synaptic ribbons relative to the base of the OHC was comparable to the initial CtBP2 immunolabelling study. This dislocation of the ribbon synapses was selective for the *Prph*KO OHCs, as CtBP2 and VAChT labeling of the IHC/ISP regions were comparable between WT and *Prph*KO cochleae.

Displacement of CtBP2-immunolabelled hair cell ribbon synapses is a primary marker of deafferentation (32) and analysis of the location of the ribbons provided an objective assessment of the disruption of the *Prph*KO type II SGN afferent synapses with the OHC. The location of the OHC pre-synaptic ribbon complexes was quantified by batch-processing WT and *Prph*KO cryosections and undertaking a blinded measurement and analysis of the distance of the CtBP2 puncta relative to the equator line of OHC nuclei in z stacks of optical sections using a 63x oil-immersion objective, with measurement of 13–57 OHC ribbons in cryosections from each of 5 WT mice (total 175 ribbons) and 6–60 ribbons from cryosections from each of 6 *Prph*KO mice (total 214 ribbons) (Figure 6C shows the ribbon distribution relative to the nucleus equator) (see also Supplementary material 8). Overall, the average CtBP2 puncta location in WT was 1.81 ± 0.24 μm below the OHC nucleus equator, compared with 0.53 ± 0.36 μm above the OHC nucleus equator for the *Prph*KO. This apical migration of the ribbons in *Prph*KO OHCs compared with the average location of the ribbon synapses in the WT OHCs was highly significant. In WT OHC, only 16.5 ± 5.4% of the CtBP2 immunopositive synaptic ribbons were located above the nucleus equator, whereas in the *Prph*KO mice this increased to 41.9 ± 3.6% (Figure 6D; *p* = 0.00313, *t*-test); indicative of disruption of synaptic integrity with the type II SGN afferent terminals in the *Prph*KO.

### Acoustically-evoked contralateral suppression in the *Prph* knockout mouse

**Figure 7:**
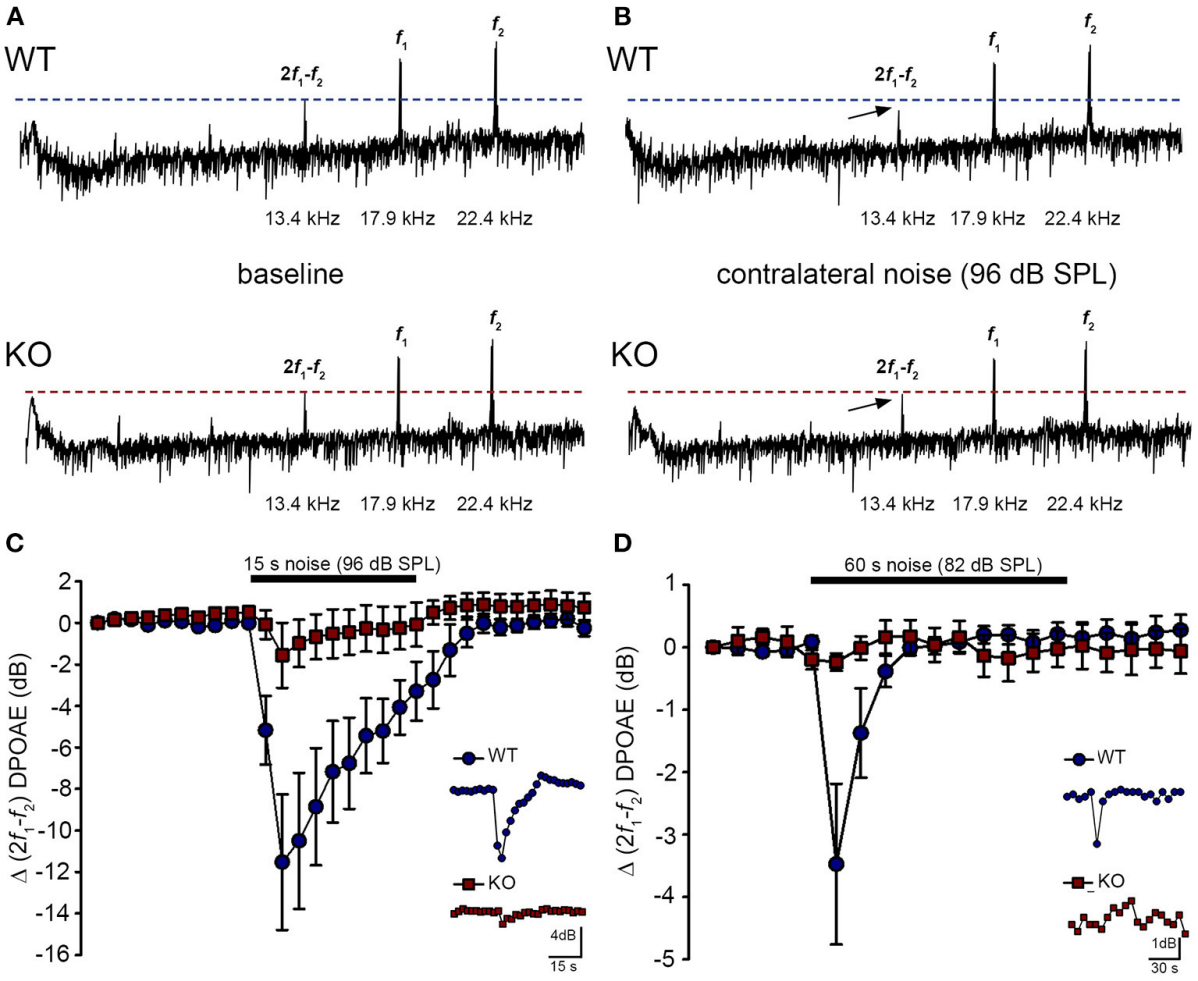
Contralateral suppression (CS) of the cubic (2f_1_-f_2_) DPOAE in wildtype (WT) and *Prph*KO mice under ketamine/xylazine/acepromazine anesthesia. **(A,B)**, Examples of fast Fourier transforms of the DPOAE in the frequency domain with WT and KO at baseline **(A,B)** with the presentation of contralateral noise (96 dB SPL, 15–25 kHz, 15 s duration, with f_1_ and f_2_ about 20 kHz using 60 dB SPL drivers). The cubic DPOAE (arrows) is diminished in the WT mouse (contralateral suppression) but is not affected in the KO mouse. **(C)** Data showing the difference in contralateral suppression with presentation of 96 dB SPL white noise in the WT mice and KO mice. Note the rapid onset to peak contralateral suppression at ~ 3 s, followed by almost complete adaptation by 15 s (unpaired *t*-test; *n* = 7 per group). **(D)** Difference in contralateral suppression between WT and KO mice during 82 dB SPL noise (60 s, about 10–17 kHz, with f_1_ and f_2_ about 28 kHz using 65 dB SPL drivers). Note the complete adaptation by ~30 s in the WT mice, whereas the DPOAE is unchanged in the KO mice (unpaired *t*-test; *n* = 8 per group).

The phenotype of loss of contralateral suppression in the *Prph*KO mice reported by Froud et al. (23) was based on measurement of quadratic DPOAE (*f*_2_-*f*_1_) amplitude changes. This was validated in the current study using the cubic (2*f*_1_-*f*_2_) DPOAE (Figures 7A–D), which is sensitive to changes in the gain of the cochlear amplifier (33, 34), as mediated by MOC efferent drive. There was no difference in the baseline sensitivity of OHC electromechanical transduction in WT and *Prph*KO mice [reflected as equivalent cubic DPOAE amplitudes with 60 dB SPL *f*_1_/*f*_2_ drivers; Figure 7A (WT upper trace compared with KO lower trace)]. In WT mice, presentation of broad band white noise at 96 dB SPL (15 s; Figure 7C) or 82 dB SPL (60 s; Figure 7D) to the left ear produced robust reductions in the cubic DPOAE recorded in the right ear (contralateral suppression), which largely adapted within 15 s. For 96 dB SPL contralateral noise, with 60 dB SPL pure tone drivers around 20 kHz in the ipsilateral ear, the peak noise-induced reduction in DPOAE in WT mice was −11.5 ± 3.0 dB (*n* = 7), measured 3 s after noise onset (Figure 7C). In contrast, only a residual contralateral suppression effect was detected in the *Prph*KO mice at this level (peak = −1.6 ± 1.6 dB; *p* = 0.003, one sample *t*-test, *n* = 7). The difference in peak contralateral suppression between WT and *Prph*KO mice with the 96 dB SPL noise presentation was therefore 9.9 dB (*p* = 0.013; unpaired *t-*test). The peak contralateral suppression in the WT mice with 82 dB SPL noise (65 dB SPL drivers around 28 kHz) was −3.5 ± 1.3 dB, measured at 6 s after noise onset (Figure 7D). There was no significant contralateral suppression in the *Prph*KO mouse group at any sample period with 82 dB SPL noise presentation (e.g., −0.2 ± 0.1 dB at 6 s, *p* = 0.379). The 3.3 dB difference in peak DPOAE suppression at this noise level between WT and *Prph*KO at 6 s was significant (*p* = 0.026, unpaired *t-*test; *n* = 8 per genotype).

### Validation of the viability of the medial olivocochlear efferent drive to the outer hair cells in the *Prph* knockout mouse by electrical stimulation

**Figure 8:**
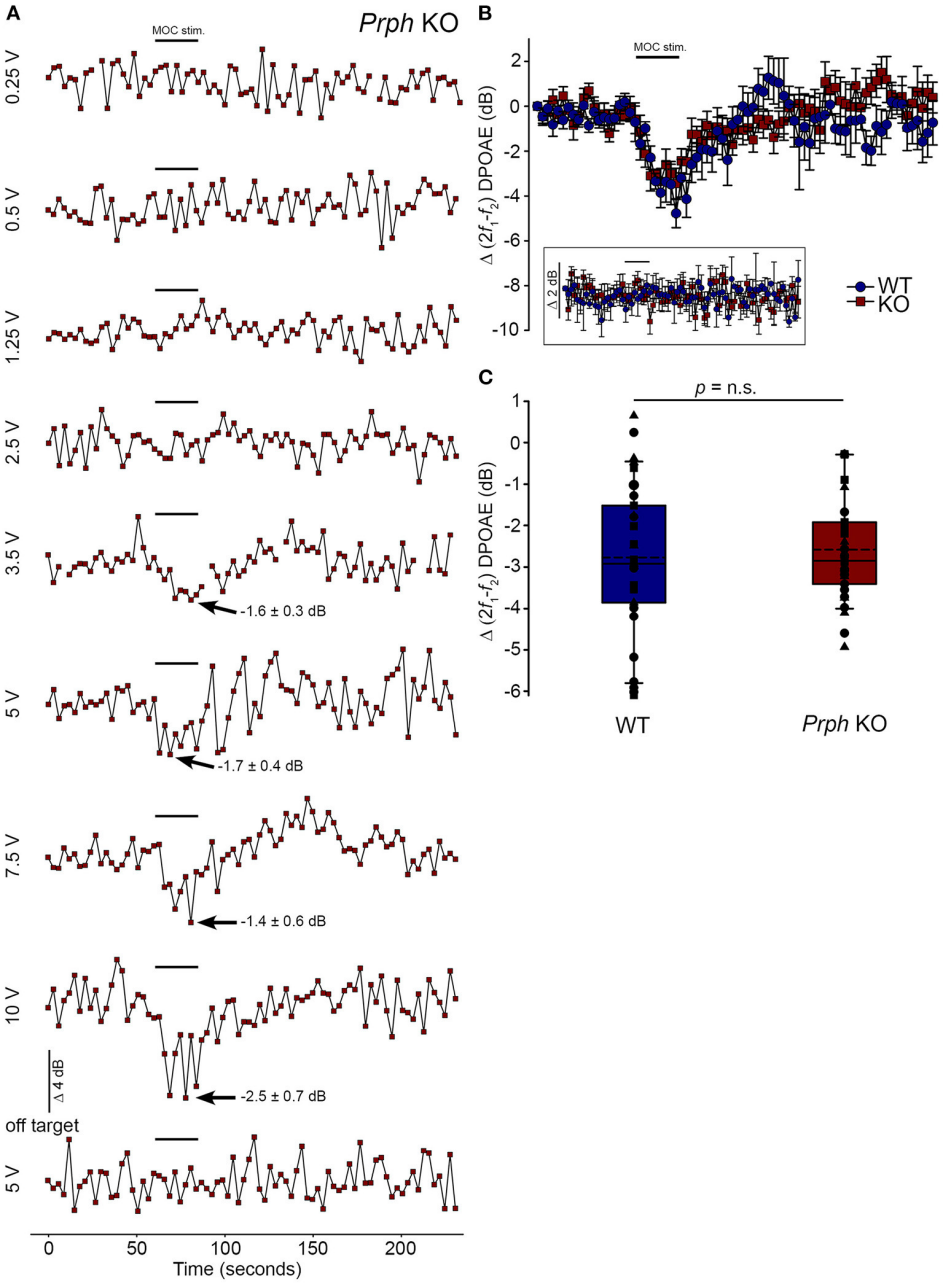
Equivalency of electrically-evoked DPOAE suppression in *Prph*KO and wildtype (WT) mice under isoflurane anesthesia. **(A)** Baseline cubic DPOAE (2f_1_-f_2_) with f_1_ and f_2_ primaries around 16 kHz at 50–55 dB SPL was established using 3 s repeated sampling for 60 s, followed by 25 s of direct electrical stimulation of the crossed olivocochlear bundle fibers on the floor of the brainstem fourth ventricle (medial olivocochlear efferent stimulation (MOC stim.), black bar; monophasic pulses, 150 μs duration, 200 Hz), and then 150 s of recovery (*Prph*KO mouse). The bottom trace (“off-target”) illustrates a “false-negative” outcome (a 2 mm rostral repositioning of the probe eliminated the suppression of DPOAE elicited with an equivalent 5 V stimulus level). Arrows indicate average contralateral suppression over the stimulus period relative to the preceding baseline average. **(B)** Overlay of the averaged electrically-evoked DPOAE suppression responses for WT and KO mice. The data show the mean and S.E.M. based on 3–5 repeated MOC stimuli for each mouse, with 25 s electrical stimulation at suprathreshold stimulus voltages (WT 5V, *n* = 3; KO 2–8 V, *n* = 3). The inset shows stability of the recordings with subthreshold stimulation (0.25–1V). **(C)** Boxplots show the equivalency of the average electrically-evoked DPOAE suppressions, with 25 and 75% boundaries and bars to 95% confidence limits. Individual data overlaid (symbols show repeats for individual mice); dashed lines show the mean; solid lines show the median. *p* = n.s. indicates *p* > 0.05, by repeated measure two-way ANOVA.

Direct electrical stimulation of the COCB efferent fiber track at the floor of the fourth ventricle was performed to eliminate the possibility that the loss of acoustically-evoked contralateral suppression in the *Prph*KO mice was due to disruption of the MOC efferent drive to the OHCs. Electrical stimulation of the MOC efferent fibers as they cross the floor of the fourth ventricle in the brainstem was used to directly compare the strength of the motor drive to the ipsilateral OHCs, assessed as cubic DPOAE suppression in WT vs. *Prph*KO mice. This physiological preparation required stable presentation of DPOAE following a craniotomy to access the fourth ventricle, and precise micro-positioning of the bipolar stimulating electrodes, with one electrode at the mid-line, ~ 0.2 mm rostral to the obex, and the second electrode positioned ~ 0.4 mm lateral, toward the ipsilateral cochlea. Transient test stimuli were used with repositioning of the probe, until suppression of the cubic DPOAE was observed. The stimulus voltage was then lowered below threshold (~3 V) and a series of recordings were obtained as the stimulus intensity was progressively stepped up to a maximum of 10 V, with 4-min recovery intervals between tests. The responses peaked within 12 s, with no substantial adaptation out to 25 s stimulation in both *Prph*KO and WT mice. Figure 8A shows an example of establishment of the stimulus threshold for electrically-evoked contralateral suppression in a *Prph*KO mouse. For statistical comparison, the amplitudes of electrically-evoked DPOAE suppression measurements at suprathreshold voltages were used (3 repeats for WT, 3–5 repeats for *Prph*KO to determine the average response for each mouse) (Figure 8B). The average reduction in DPOAE amplitude during the 25 s stimulus from these trials was then used for statistical comparison between genotypes (Figure 8C). A repeated measures two-way ANOVA showed that the sensitivity of the electrically-evoked contralateral suppression was equivalent between *Prph*KO and WT mice (WT = −2.7 ± 0.3 dB, *n* = 3; KO = −2.6 ± 0.2 dB, *n* = 3; *p* = 0.534). The need for precise positioning of the stimulus probe to achieve an electrically-evoked change in DPOAE is demonstrated in Figure 8A, where a repositioning of the probe “off-target” eliminated the DPOAE suppression. There was no difference in the amplitudes of the baseline cubic DPOAE in the WT and *Prph*KO cochleae, WT average = 17.1 ± 0.3 dB above noise-floor; KO = 16.1 ± 0.2 dB; *p* = 0.078; unpaired *t-*test).

### Loss of otoprotection from noise-induced hearing loss in *Prph* knockout mice

**Figure 9:**
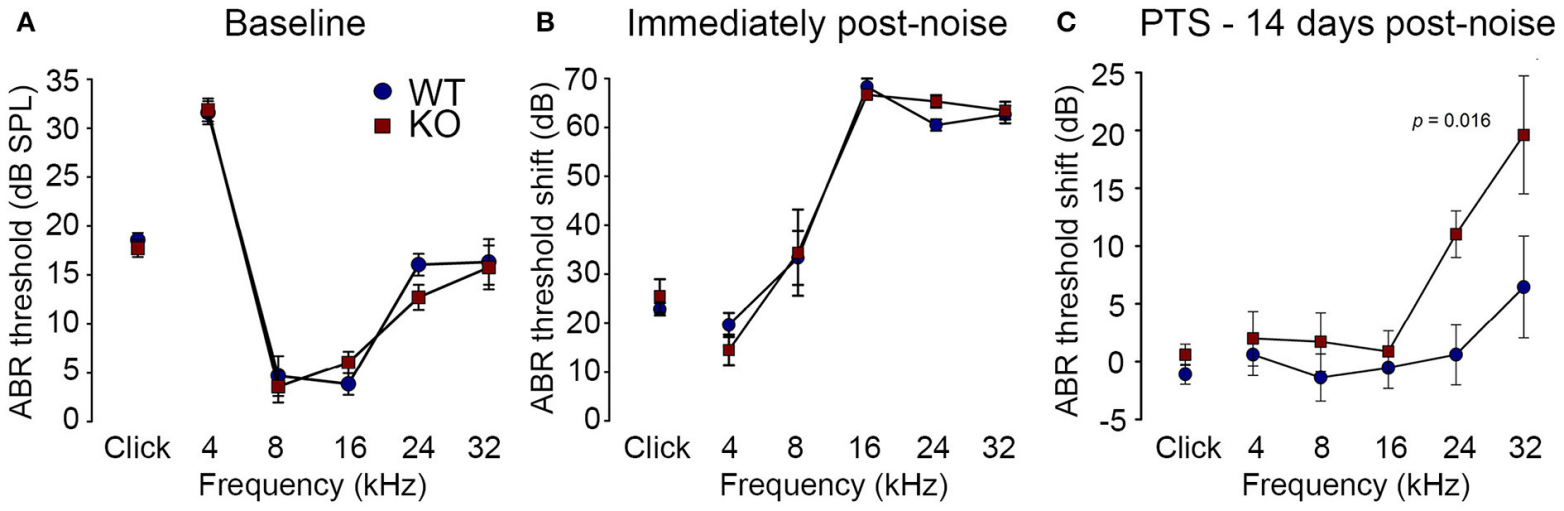
*Prph*KO mice are vulnerable to noise-induced high frequency hearing loss. **(A)** Hearing sensitivity was equivalent in KO and wildtype (WT) mice based on auditory brainstem response (ABR) thresholds; SPL sound pressure level (*n* = 9 per group). **(B)** There was no significant difference in ABR threshold shifts immediately following exposure to 1 h of 108 dB SPL white noise (4–32 kHz; open-field) between WT and KO mice. **(C)** Permanent threshold shifts were determined 2 weeks post-noise. While WT mouse thresholds returned to pre-noise levels, KO littermates had significant permanent threshold shifts at frequencies above 16 kHz (ANOVA). Grubbs' test (GraphPad) excluded one data point as an outlier for *Prph*KO 24 kHz. Ketamine/xylazine/acepromazine anesthesia (see also Supplementary materials 9–11).

Reduction of OHC electro-mechanical transduction by the MOC efferent pathway is otoprotective (14, 35). For example, surgical ablation of the COCB at the floor of the fourth ventricle in cats increased noise-induced threshold shifts (36) and over-expression of the OHC α9 nicotinic acetylcholine receptor subunit in mice confers protection to noise-induced hearing loss (NIHL) (37). Here we investigated the effect of disruption of the OHC–type II SGN sensory input arising from knockout of *Prph* in this key aspect of hearing homeostasis. The vulnerability to 1 h of acute white noise (4–32 kHz) at 108 dB SPL was assessed in WT and *Prph*KO mice by ABR (Figure 9). WT and *Prph*KO mice had equivalent pre-noise ABR thresholds for click and tone-pip stimuli (4–32 kHz) (Figure 9A), which indicates that disruption of the type II SGN sensory drive does not affect hearing sensitivity in a nominally quiet environment. Pre-noise click input-output functions were also equivalent (click ABR: 0.062 ± 0.009 μV/dB WT; 0.078 ± 0.011 μV/dB *Prph*KO; *p* = 0.277, *t-*test; *n* = 9 per group). ABR threshold shifts immediately after noise exposure were comparable between the WT and *Prph*KO mice, with the greatest changes (~60–70 dB) observed from 16–32 kHz (Figure 9B). Permanent hearing loss (permanent threshold shift–PTS), was determined by re-measurement of ABR thresholds 2 weeks after noise exposure. While the WT mice did not exhibit any significant PTS, with thresholds returning to pre-noise baseline, the *Prph*KO mice exhibited high frequency hearing loss, with PTS of 10.9 ± 2.0 dB at 24 kHz and 19.4 ± 5.1 dB at 32 kHz [*p* < 0.016; Two-way RM ANOVA; *n* = 9 per genotype (Figure 9C)]. Data distribution from individual animals at 24 kHz and 32 kHz shown in boxplots (Supplementary material 10). The Power of the two-way ANOVA analysis of genotype effect on noise-induced hearing loss was 0.655 with alpha = 0.05. The ABR input/output functions for neural recruitment of the WT mice, and *Prph*KO mice 2 weeks post-noise, demonstrated equivalency at frequencies where the *Prph*KO mice exhibited significant threshold shifts (Supplementary material 11). The average slopes for 24 kHz were: WT = 0.032 ± 0.002 μV/ dB; *Prph*KO = 0.038 ± 0.005 μV/ dB. 32 kHz slopes were: WT = 0.032 ± 0.003 μV/ dB; *Prph*KO = 0.038 ± 0.005 μV/ dB (two-way RM ANOVA for genotype comparison, *p* > 0.05). Supporting comparable ABR input/output functions; cubic DPOAE (8 kHz, 12 kHz, 16 kHz, 24 kHz, and 32 kHz) at 2 weeks post-noise showed no significant differences in thresholds (respective difference of means: 2.22 dB, 1.11 dB, 4.44 dB, 5.00 dB, 8.33 dB); *p* > 0.05 two-way RM ANOVA; input/output functions (*p* > 0.05 two-way RM ANOVA); consistent with preservation of OHCs and potential type I afferent synaptopathy underlying the ABR PTS.

The potential for noise-induced hair cell loss and synaptopathy in the basal cochlea region, anatomically mapped to the observed high frequency PTS (38), was examined in cryosections from 3 WT and 2 *Prph*KO mice following the 2 week post-noise PTS assessment. Myosin 7A immunolabelling confirmed OHC and IHC preservation (Supplementary material 12). Similarly nominal pre-synaptic ribbon presentation at the base of the IHCs in WT and *Prph*KO noise-exposed tissue was maintained based on CtBP2 immunofluorescence (Supplementary material 12). The pre-synaptic ribbons maintained close juxtaposition to post-synaptic GluA2 type I afferent terminals at the IHC, consistent with preserved function (Supplementary material 12). Irregular localization of *Prph*KO OHC CtBP2 immunopositive ribbons was anticipated by the phenotype [compare (Supplementary material 12)]. The lack sensitivity in identifying putative hair cell cyotopathology is consistent with the modest discrete PTS generated by the noise challenge (39, 40).

## Discussion

These studies provide strong evidence that at loud sound levels, the OHC–type II SGN afferent input to the cochlear nucleus contributes to the activation of the MOC efferent feedback control of the cochlear amplifier to confer protection from noise-induced hearing loss. Hearing sensitivity (threshold ABR and DPOAE amplitude) is equivalent for WT and *Prph* deficient mice. However, in the *Prph*KO, acoustically-evoked contralateral suppression was absent at 82 dB SPL noise (just above the threshold for detectable WT contralateral suppression), and was ~ 10 dB less than WT using high level (96 dB SPL) noise (where 3 dB represents a halving of intensity). These measurements were undertaken at 20–28 kHz, probing the middle region of the mouse cochlea (41). The region exhibits profound type II SGN dendrite dysmorphology in the *Prph*KO cochlea (Figure 3B), with only residual outer spiral fibers in evidence, and OHC synaptic ribbons significantly displaced.

Our initial NF200 immunolabelling in adult *Prph*KO cochlea identified the significant reduction in the number of outer spiral fibers (23). This phenotypic characteristic was subsequently disputed by Maison et al. (11), who reported a normal OSB structure, and the stereotypical localization of the OHC CtBP2-immunopositive pre-synaptic ribbons, in their *Prph*KO mice. In the present study, we validated the characterization of the abnormal OSB fiber phenotype in our *Prph*KO mouse model using NF200, β-III tubulin, and parvalbumin immunofluorescence modalities. CtBP2 immunolabelling of the OHC pre-synaptic ribbons showed evident disruption (apical drift) from the ordered basal localization in the WT, consistent with deafferentation (32). This observation was statistically validated by the blinded study that measured the position of several hundred WT and *Prph*KO CtBP2 positive pre-synaptic ribbons with respect to OHC nucleus equator, across 5 WT and 6 PrphKO mice (Supplementary material 8). The finding of loss of tethering of the pre-synaptic ribbons in the *Prph*KO OHC supports our previous characterization of the (post-synaptic) afferent bouton structure using serial blockface scanning electron microscopy (23), (Supplementary Figure 2 and Supplementary Table 1). In that study no type II SGN afferent boutons could be identified in synaptic contact with *Prph*KO OHCs, compared to 1–2 reconstructed afferent terminals per WT OHC; consistent with other mouse data (19, 42). In the same WT and *Prph*KO samples, ~ 2.3 MOC efferent boutons were rendered per OHC for both genotypes (23). Outside of a potential tissue genotyping mismatch, it does not seem possible to reconcile the structural anomaly in the type II afferent innervation of the OHCs which we have detailed across our studies, with the Maison et al. (11) report. In addition to Froud et al. (23), we have also previously demonstrated that the knockout of peripherin expression altered regulation of type II afferent neurite growth in an *in vitro* neonatal cochlear spiral ganglion explant model (43); a finding congruent with the anomalous outer spiral bundle structure in the adult *Prph*KO mice.

Disruption of type II SGN afferent neurites in the *Prph*KO cochlea is consistent with evidence that the type III intermediate filament protein peripherin supports axon growth and synaptic consolidation, particularly in sensory fibers of the peripheral nervous system (44–46). *Prph knockout* was originally reported to cause selective loss of small diameter unmyelinated nociceptor dorsal root ganglion (DRG) fibers, while sparing the majority of peripherin-positive DRG neurons (28). This variation in impact of *Prph* gene deletion has been linked to co-regulation of complementary type IV intermediate filaments, α-internexin and neurofilament proteins (particularly NF-L) which jointly contribute to nerve fiber outgrowth, branching and synapse formation (28, 47, 48). On this basis, the selective impact of *Prph*KO on the mouse cochlear type II SGN innervation of the OHCs may reflect insufficiency of compensation from co-expressed neurofilaments. In the brain, *Prph* expression is largely constrained to cranial nerve sensori-motor pathways, such as the mesencephalic trigeminal nucleus (49). Injury stimulates *de novo* expression of peripherin in the brain, leading to aggregation of the protein and neurodegeneration (50). Upregulation of *Prph* expression contributes to injury repair responses in the peripheral nervous system, for example, being linked to axon regrowth and sprouting following sciatic nerve crush (51). While largely conserved across species, peripherin expression does exhibit spatiotemporal variations across tissues likely associated with variations in promoter and regulatory domains (46). In the rat cochlea, peripherin expression overlaps with type I SGN for several days beyond birth (52), while in the mouse, peripherin is strongly biased to the unmyelinated type II SGN, over type I SGN from ~ E18 onwards, and is most strongly represented in those OSB fibers during early post-natal neurite extension and synaptic consolidation across the first post-natal week (24, 25, 53).

*In vivo* electrophysiological studies of type II SGN are limited, but suggest that these small, unmyelinated neurons have slow conduction velocities, high thresholds and relative insensitivity to sound stimulation. Robertson (54) delivered horseradish peroxidase during intracellular high-impedance microelectrode recordings of SGN somata in the guinea pig Rosenthal's canal to definitively label an outer spiral fiber projecting to basal OHCs. Unlike type I SGN, which project as radial fibers to IHCs and respond to sound stimulation, this single labelled type II SGN was “silent” (54). The more definitive data of Brown (21) is from 19 putative guinea pig type II units recorded with high impedance glass microelectrodes. These unlabelled, but presumed type II SGN, were characterized by longer antidromic response latencies than type I SGN units. Some responded to acoustic stimulation, with the most sensitive threshold at 80 dB SPL, matching the current contralateral suppression paradigm. Units with the longest antidromic latencies were generally unresponsive to sound. In a subsequent guinea pig study (55), additional SGN recordings from “silent” units with long antidromic stimulus latencies were evaluated, but the identity and integrity of these cells was questioned.

Additional studies of type II SGN physiology have been performed *in vitro*. *In situ* whole-cell patch-clamp analysis in a rat neonatal cochlear slice preparation showed comparable recruitment of action potentials in type I and type II SGN, with an A-type inactivating K^+^ channel conductance prominent in the type II SGN, with reduced AMPA-type glutamate receptor conductance, but comparable ATP-activated inward currents (20). Type II SGN had significantly depolarized resting potentials compared with type I SGN, which may explain the limited ability to record action potentials from these neurons *in vivo* with microelectrodes. Under current-clamp, isolated mouse type II SGN exhibited region-dependent variation in firing properties similar to those of type I SGN (56). Patch-clamp recordings of neonatal rat type II SGN terminal processes provided definitive evidence of OHC glutamatergic neurotransmission and purinergic neuromodulation, including spontaneous excitatory post-synaptic potentials/currents (EPSP/EPSC) (57). Glutamatergic transmission appeared weaker than that of IHC–type I SGN synapses, based on the limited number of spontaneous EPSC events. These studies were extended and showed synaptic transmission *via* GluA2-containing AMPA receptors evoked by local application of K^+^. The data are consistent with a requirement for integrated summation of synaptic transmission from ~ 6 OHCs to reach action potential threshold, sustained by an outer spiral fiber length constant beyond 1 mm, and supported by Na_v_1.6 voltage-gated Na^+^ channels (5, 58, 59).

There is overlapping distribution of type I and type II spiral ganglion afferent input to the cochlear nucleus. Both type I and type II SGN have branching termination in the dorsal and ventral cochlear nuclei, with type II SGN having considerable additional input to the cochlear nucleus granule cell region, a feature lacking in type I SGN (17). In the small cell region in the posteroventral cochlear nucleus region (PVCN) immediately adjacent to the granule cell domain (60), electron microscopy localized type II synapses on stellate cells (61). This region also receives high-threshold type I auditory nerve fibers (62), has high-threshold auditory neurons (63), and projects to the olivocochlear neurons (64, 65). Darrow et al. (66) used dye injections into the mouse cochlea for retrograde labeling of contralateral MOC efferent neurons in the ventral nucleus of the trapezoid body, alongside anterograde labeling from the PVCN that resolved planar stellate cells synapsing onto those MOC somata and dendrites; local PVCN ablation reduces MOC reflex suppression (10). Alongside this, studies across species have identified reciprocal connectivity between the MOC neurons and the cochlear nuclei, reflecting complexity of afferent and efferent circuitry regulating the MOC reflex (65, 67, 68). A notable finding in the gerbil (69) and mouse (70) is that MOC efferent collaterals project to the cochlear nucleus granule cell region, a selective convergence with type II SGN afferent input. In support of the *in vivo* electrophysiological findings for type II SGN functionality, using noxious noise levels Flores et al. (71) reported increase in *cFos* signal in the cochlear nucleus granule cell region in *VGlut3* KO mice, which lack IHC–type I SGN transmission. This finding has been advanced by a more definitive study which selectively modulated OHC glutamatergic neurotransmission to demonstrate functional sensory drive by type II SGN afferents to the granule cell region of the cochlear nucleus spanning moderate (80 dB SPL) to damaging (115 dB SPL) sound levels (16).

It is clear from the present study that the loss of acoustically-evoked contralateral suppression in the *Prph*KO mice occurs upstream of the superior olivary complex, in the afferent supply to the reflex, since the COCB remains functional. This is counter to the Maison et al. (11) *Prph*KO study, which did not observe the type II SGN morphological disruption, and where a negative result for electrically-evoked COCB suppression of the DPOAE in two *Prph*KO mice led to an inference that loss of contralateral suppression likely arose from failure of the efferent arm of the circuit. The present study verifies type II SGN synapse disruption and dendrite loss in the *Prph*KO mouse line. With the strong evidence (16) that type II SGN are acoustically active at moderate and high sound levels, the loss of contralateral suppression in the *Prph*KO mice seems likely due to the type II SGN synapse disruption. A radical change in drive from the IHC–type I afferent sensory input in the *Prph*KO mouse appears less likely, since hearing sensitivity is maintained. The possibility of selective loss of a subset of high threshold afferent input to the MOC region such as the the PVCN small cells which connect reciprocally is also a possibility, but there is no evidence for peripherin expression in the cochlear nucleus (49). Other alternatives are possible. A small number of the MOC tunnel crossing fibers were reported as peripherin immunopositive in wildtype mice (11), although we show here that the electrically-evoked MOC drive for DPOAE suppression was equivalent in the *Prph*KO. In the cochlea, type II SGN fibers make terminal arborizations with Deiters' and Hensen's cells, particularly in the apical region, although it is uncertain whether these reflect synaptic couplings (72). Reciprocal transmission between type II SGN afferents and the OHCs, evident from electron microscopy studies in cat and primates, could also contribute to local integration of OHC sensory transduction (73). There are also synaptic varicosities between type II SGN afferents and the MOC efferent terminals (74) which may support GABAergic transmission as part of a reciprocal microcircuit in the early post-natal period in rats (75). Changes in these cochlear connections could potentially be a factor.

Any of these potential mechanisms demonstrate a critical role for peripherin in afferent feed to contralateral suppression. However, in the absence of evidence supporting alternatives, we feel that parsimony favors loss of OHC–type II SGN input as the root cause of loss of contralateral suppression with 82–96 dB SPL broadband noise range tested here. This postulate is arguably most strongly supported by a complementary mouse study that selectively reduced IHC–type I afferent input to the cochlear nucleus by treating the cochlear round window membrane with ouabain, causing ~ 90% reduction in the ABR wave I amplitude, but preserved contralateral suppression using 76 dB SPL broadband noise (15).

Given the long-standing evidence that type I SGN afferent input drives the MOC efferents, based on near-equivalency of single fiber thresholds and tuning characteristics (12, 13), the association here of disruption of type II SGN input and loss of contralateral suppression suggests that the broad reduction of the cochlear amplifier by the COCB at higher noise levels, captured by DPOAE, may require the combined drive of both type I SGN and type II SGN input, with the unexpected finding that type I SGN drive alone may fall short with regard to the role of the MOC feedback circuit for protection of the hair cells and their synapses from acoustic overstimulation. It is well established that MOC efferent feedback is critical to the long-term protection of the cochlear hair cells and their afferent innervation (4). For example, chronic moderate (84 dB SPL) noise exposure caused increased type I SGN synaptopathy in mice when the COCB was sectioned at the floor of the fourth ventricle (76). MOC efferent–mediated protection from NIHL has been confirmed in mice using selective genetic manipulation of the OHC nicotinic cholinergic receptor (nAChR). As noted above, a gain of function mutation of the α9 nAChR subunit confers heightened resistance to NIHL (77, 78), as does α9 nAChR subunit overexpression (37). Knockout of the α9 nAChR subunit enhances NIHL (78). That study in the α9 nAChR KO utilized a lower frequency noise band (1–16 kHz, 100 dB SPL, 1 h, open field, with ketamine/xylazine anesthesia) than the present study and achieved higher broad sustained threshold shifts (averaging up to 35 dB compared with no PTS in WT mice). While no hair cell loss was detectable, a quantitative analysis of CtBP2 and GluA2 IHC synapse immunofluorescence across hundreds of IHCs in whole-mount preparations resolved significant synaptopathy. Given that our ABR PTS shifts were smaller and our study was not powered to quantitatively assess synaptic integrity, the NIHL associated with the *Prph*KO–mediated disruption of the MOC reflex most likely stems from similar synaptopathy.

These findings provide a clear imperative for development of more sophisticated models for selective manipulation of the two cochlear afferent populations to probe the sensory drive of the MOC efferent control of the cochlear amplifier. This challenge may be met in part by leveraging the use of *Prph* gene targeting by integrating Bacterial Artificial Chromosomes (BAC) in transgenic mice. For example, a mouse line integrating a 150 kb BAC containing the human *PRPH* gene and associated promoter and regulatory non-coding regions engineered to generate a (functional) peripherin fusion protein with enhanced green fluorescent protein (*PRPH*-eGFP) (46) has proved to be an effective type II SGN afferent biomarker, with strong correlation to native mouse cochlear peripherin immunolabelling (53). While crossing of this mouse model to the current *Prph*KO mouse line would retain peripherin protein translation (from the BAC *PRPH* expression); modification of this BAC, or a similar mouse *Prph* BAC as developed by the Gene Expression Nervous System Atlas (GENSAT) program (79), to integrate a conditional human diptheria toxin receptor (hDTR) transgene element into the *Prph* reading frame, may allow selective ablation of the adult cochlea type II SGN, extending functional insight into the role(s) of the OHC–type II SGN afferent pathway in hearing.

In summary, in the *Prph*KO mouse model, we demonstrate clear association between the disruption of OHC–type II SGN sensory input, near-elimination of MOC efferent–mediated contralateral suppression at moderate to high sound levels, and reduction in otoprotection against NIHL. The findings support the contribution of cochlear OHC-type II SGN afferents to the sensory drive of the MOC efferent suppression of the cochlear amplifier that confers noise-induced hearing loss protection.

## Data availability statement

The original contributions presented in the study are included in the article/Supplementary material, further inquiries can be directed to the corresponding author/s.

## Ethics statement

The animal study was reviewed and approved by UNSW Sydney Animal Care and Ethics Committee.

## Author contributions

JC, KP, GH, AR, and J-PJ contributed to the conception and design of the study. JC, KP, JP, and GH undertook the physiological studies. JC, KP, CP, GJ, DR, AR, and GH performed the histological and imaging studies and structural analysis. GH, AR, and DR contributed to the funding. All authors contributed to the data validation and presentation, production of the manuscript, and approved the submitted version.

## Funding

This work was supported by Australia grants from the National Health & Medical Research Council (NHMRC) APP1052463, APP1189113, and APP1188643 & U.S. Department of Veterans Affairs grants BX001205 and RX002704. The authors declare that this study also received funding from Alan and Lynne Rydge to the Hearing Research Lab at the Garvan Institute. They were not involved in the study design, collection, analysis, interpretation of data, the writing of this article or the decision to submit it for publication.

## Conflict of interest

Author AR is a co-founder of Otonomy Inc., serves as a member of the Scientific Advisory Board, and holds an equity position in the company. The UCSD Committee on Conflict of Interest has approved this relationship. Otonomy, Inc. played no part in the research reported here. The remaining authors declare that the research was conducted in the absence of any commercial or financial relationships that could be construed as a potential conflict of interest.

## Publisher's note

All claims expressed in this article are solely those of the authors and do not necessarily represent those of their affiliated organizations, or those of the publisher, the editors and the reviewers. Any product that may be evaluated in this article, or claim that may be made by its manufacturer, is not guaranteed or endorsed by the publisher.
