# Supplementary material for: Noise-induced hearing loss vulnerability in type III intermediate filament peripherin gene knockout mice
Source: Front Neurol. 2022 Sep 26;13:962227. doi: 10.3389/fneur.2022.962227 (PMC9549866; doi:10.3389/fneur.2022.962227)
Supplement: Supplementary file 1 [file Presentation_1.pdf]

Supplementary Material

Cederholm *et al*. Frontiers in Neurology - Neuro-Otology. **Noise-induced hearing loss vulnerability in type III intermediate filament peripherin gene knockout mice**

Doi: 10.3389/fneur.2022.962227

Supplementary Material:

Supplementary Material 1: Peripherin knockout genotyping and validation

Supplementary Material 2: Video 1(*Filename: Cederholm et al. SIvideo1.wmv & Cederholm et al. SIvideo1.wmv_title.jpg)*

Supplementary Material 3: Figure 2 supplement 1

Supplementary Material 4: Video 2 (*Filename: Cederholm et al. SIvideo2.wmv & Cederholm et al. SIvideo2.wmv_title.jpg)*

Supplementary Material 5: Video 3 (*Filename: Cederholm et al. SIvideo3.wmv & Cederholm et al. SIvideo3.wmv_title.jpg)*

Supplementary Material 6: Figure 4 supplement 1

Supplementary Material 7: Figure 6 supplement 1

Supplementary Material 8: Figure 6 Source Data

Supplementary Material 9: Figure 9 Source Data

Supplementary Material 10: Figure 9 supplement 1

Supplementary Material 11: Figure 9 supplement 2

Supplementary Material 12: Figure 9 supplement 3

Cederholm *et al*. Frontiers in Neurology - Neuro-Otology. **Noise-induced hearing loss vulnerability in type III intermediate filament peripherin gene knockout mice**

Doi: 10.3389/fneur.2022.962227

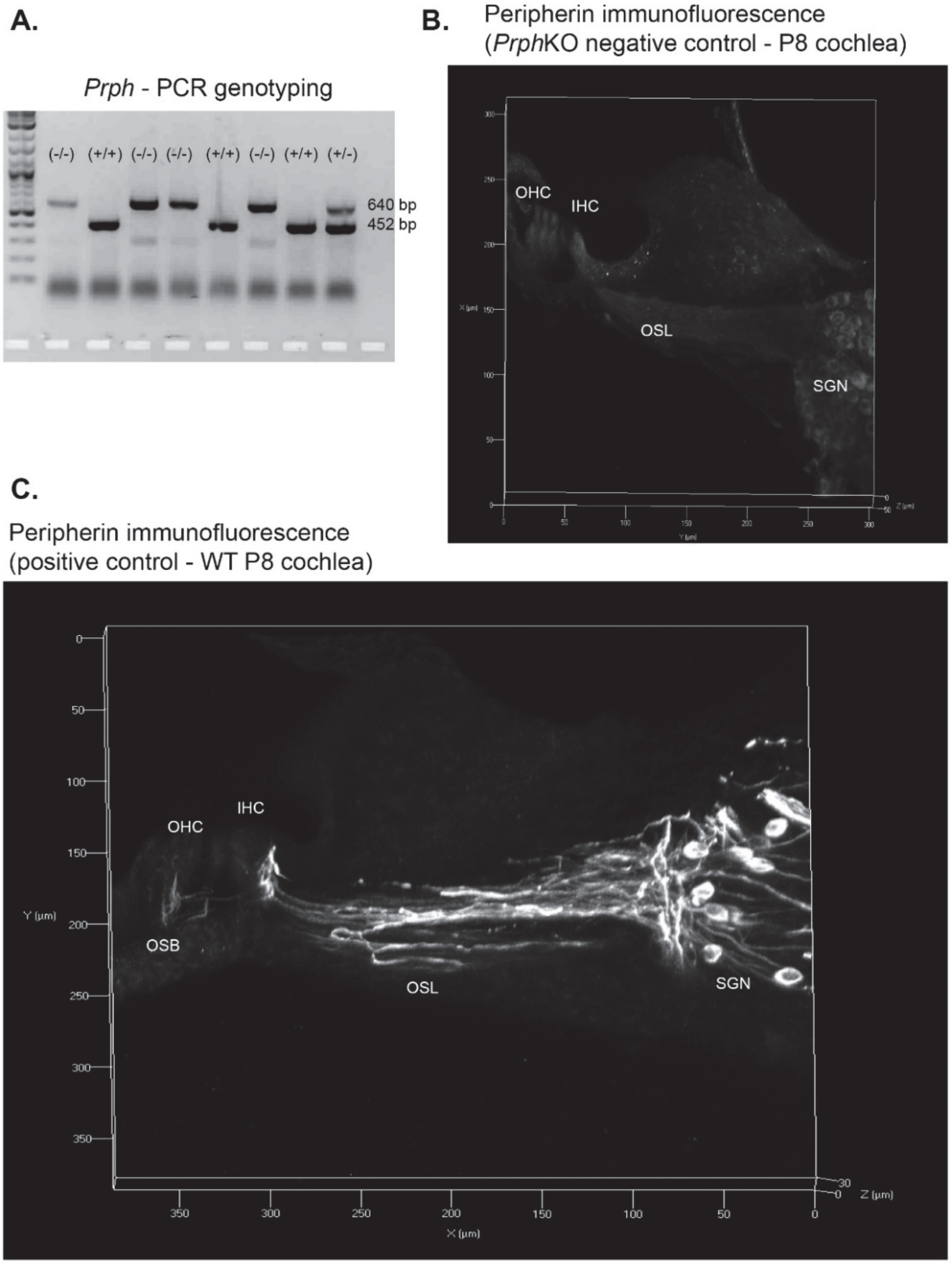

Supplementary Material 1: Peripherin knockout mouse line genotyping and validation through cochlear immunofluorescence . *A., Agarose gel electrophoresis of PCR amplicons delineates wildtype (+/+; 452 bp), heterozygous (+/-; 640 bp & 452 bp) and knockout (-/-; 640 bp) genotypes. B., The knockout genotype was confirmed by the absence of immunofluorescence labelling using post-natal day 8 (P8) PrphKO mouse cochlea. C. Image of type II spiral ganglion afferent neuron somata and their peripheral neurites which form the outer spiral bundles (OSB) in wild type P8 cochlea (anti-peripherin antibody batch-processed with the PrphKO tissue). SGN, spiral ganglion neurons; OSL, osseous spiral lamina; OHC, outer hair cells; IHC, inner hair cells. B and C are 3D projections of confocal optical sections acquired from 50 µm cryosections immunolabelled with the anti-peripherin antibody and alexa488 fluorescent secondary antibody.*

Cederholm *et al*. Frontiers in Neurology - Neuro-Otology. **Noise-induced hearing loss vulnerability in type III intermediate filament peripherin gene knockout mice**
Doi: 10.3389/fneur.2022.962227

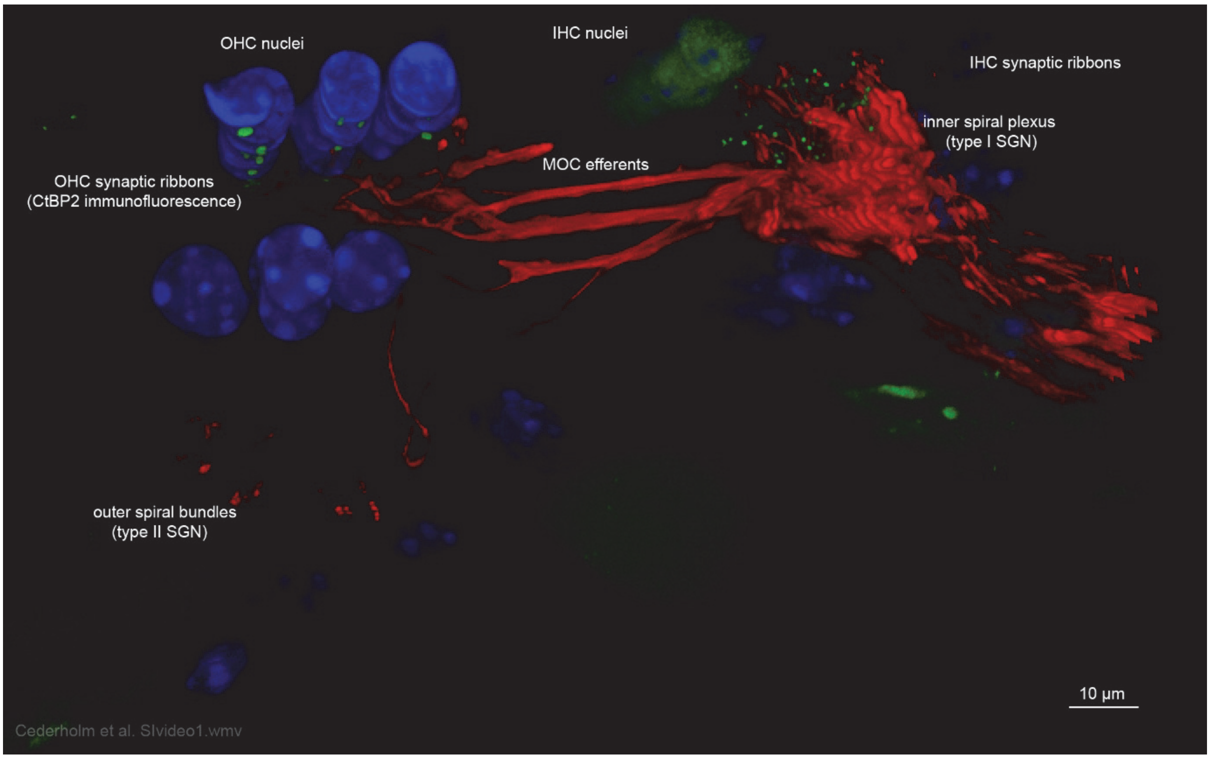

Supplementary Material 2: Video 1. Projected confocal image stack of CtBP2 immunofluorescence (green) highlights the regular synaptic ribbon structure at the base of the three rows of outer hair cells, with one or two discrete puncta beneath the nucleus, compared with the dense synaptic ribbon complex associated with the basolateral membrane of each inner hair cell (WT cochlear organ of Corti). *Type II spiral ganglion afferents (type II SGN) form the outer spiral bundle beneath the Deiters' cells (NF 200 immunofluorescence – red) and project apically to the OHCs (upper left). This neural labelling includes the dense inner spiral plexus of the type I spiral ganglion neurite innervation (type I SGN) of each inner hair cell (IHC) – juxtaposed to the dense ribbon complex (upper right). The NF 200 immunofluorescence also extends to the dense matrix of medial olivocochlear (MOC) efferent fibres crossing the tunnel of Corti to innervate the outer hair cells. This projection extends the visualisation of Figure 1C.* ***Filename: Cederholm et al. SIvideo1.wmv***

Cederholm *et al*. Frontiers in Neurology - Neuro-Otology. **Noise-induced hearing loss vulnerability in type III intermediate filament peripherin gene knockout mice**

Doi: 10.3389/fneur.2022.962227

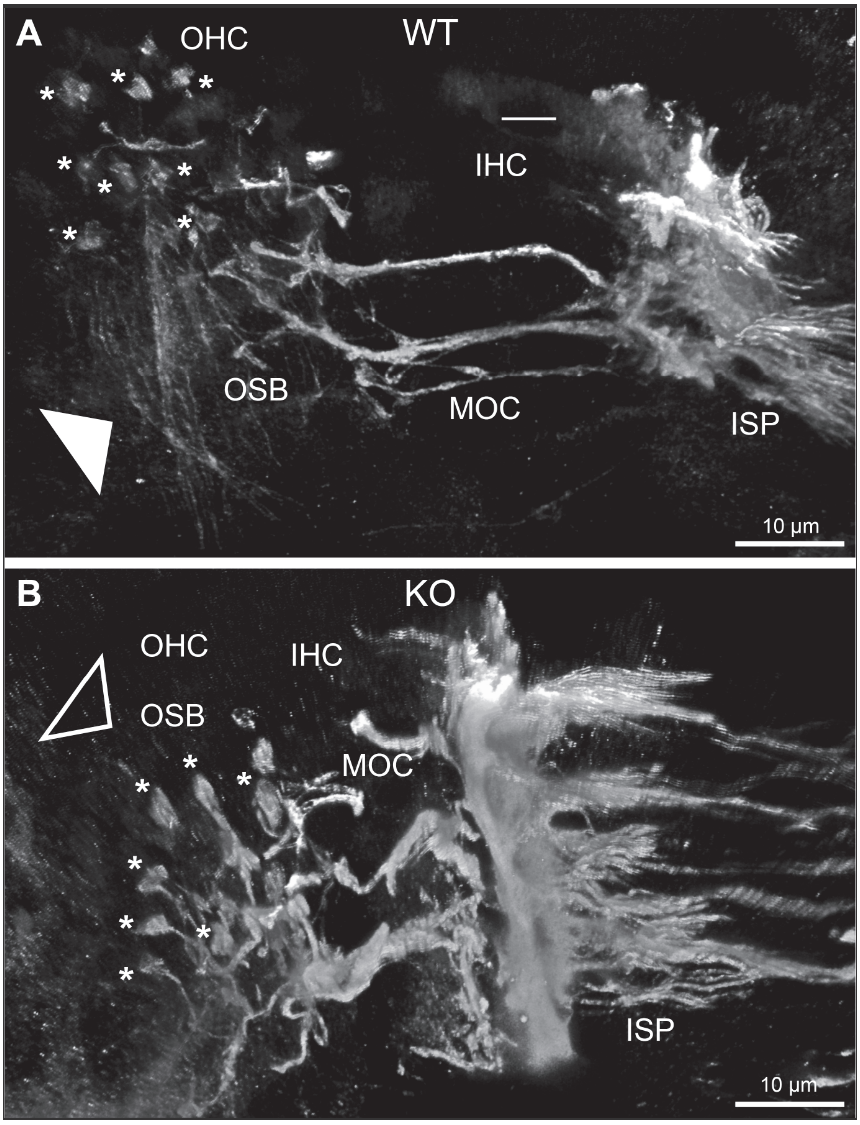

Supplementary Material 3: Figure 2 supplement 1. Disruption of outer spiral bundle structure (OSB; type II spiral ganglion neuron dendrites (type II SGN)) in mid - cochlear region of the Prph knockout (KO) resolved using β-III tubulin immunofluorescence. *Confocal transparent-mode 3D reconstructed images of batch-processed WT and PrphKO cryosections with the basilar membrane surface oriented uppermost to optimally delineate the OSB.* ***A****, In the WT cochlea, the outer spiral fibres (type II SGN) within the OSB (arrowhead) run vertically below the three rows of outer hair cells (OHCs). The medial olivocochlear (MOC) efferent fibers project horizontally from the inner spiral plexus (ISP) region, to the OHC region, where they branch to form large synaptic boutons (*) at the base of multiple OHCs.* ***B****, In the PrphKO image, the OSB are not evident (open arrowhead), while equivalent MOC efferent fiber projections extend to the OHCs and form synaptic boutons (*). IHC, inner hair cells.*

Doi: 10.3389/fneur.2022.962227

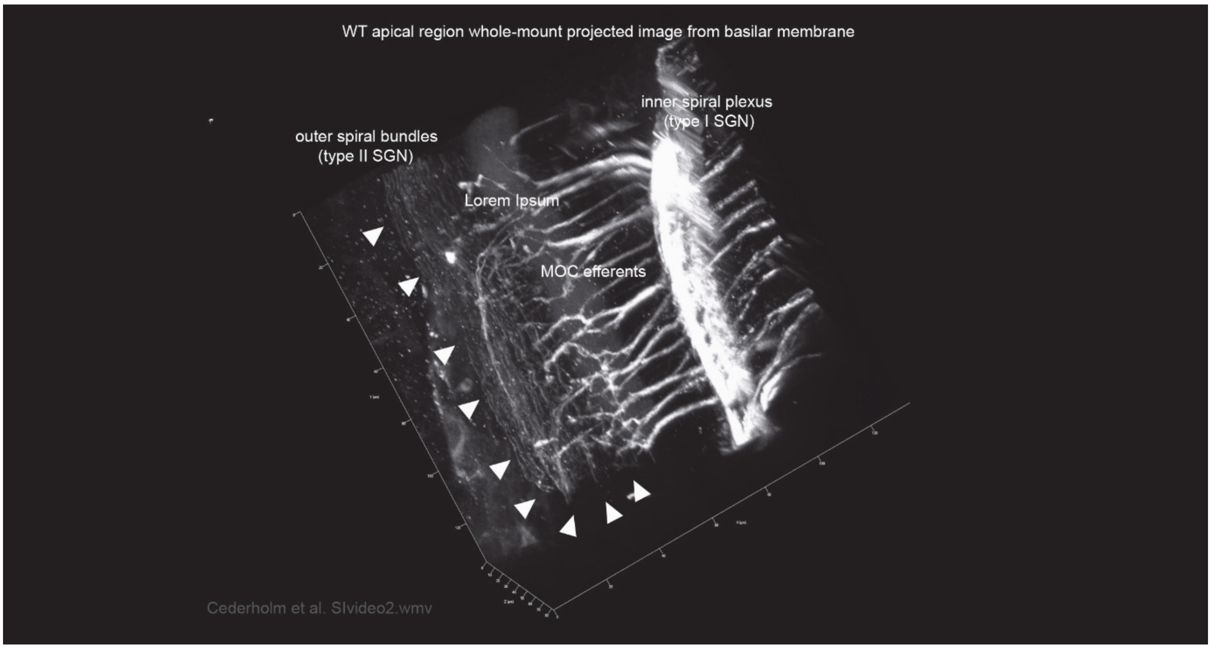

Supplementary Material 4: Video 2. β-III tubulin immunofluorescence of innervation of the organ of Corti in the apical region of the wildtype mouse. *Type II spiral ganglion afferents (type II SGN) form outer spiral bundles of small diameter unmyelinated neurites that run beneath the three rows of outer hair cells. Whole-mount maximum intensity projection of a confocal image stack (48 µm) imaged from the basilar membrane surface. The inner spiral plexus of type I spiral ganglion neurites (type I SGN) innervate the single row of inner hair cells. The medial olivocochlear (MOC) efferent fibres are shown crossing the tunnel of Corti to innervate the outer hair cells. This projection extends the visualization of Figure 3A.* ***Filename: Cederholm et al. SIvideo2.wmv***

Cederholm *et al*. Frontiers in Neurology - Neuro-Otology. **Noise-induced hearing loss vulnerability in type III intermediate filament peripherin gene knockout mice**

Doi: 10.3389/fneur.2022.962227

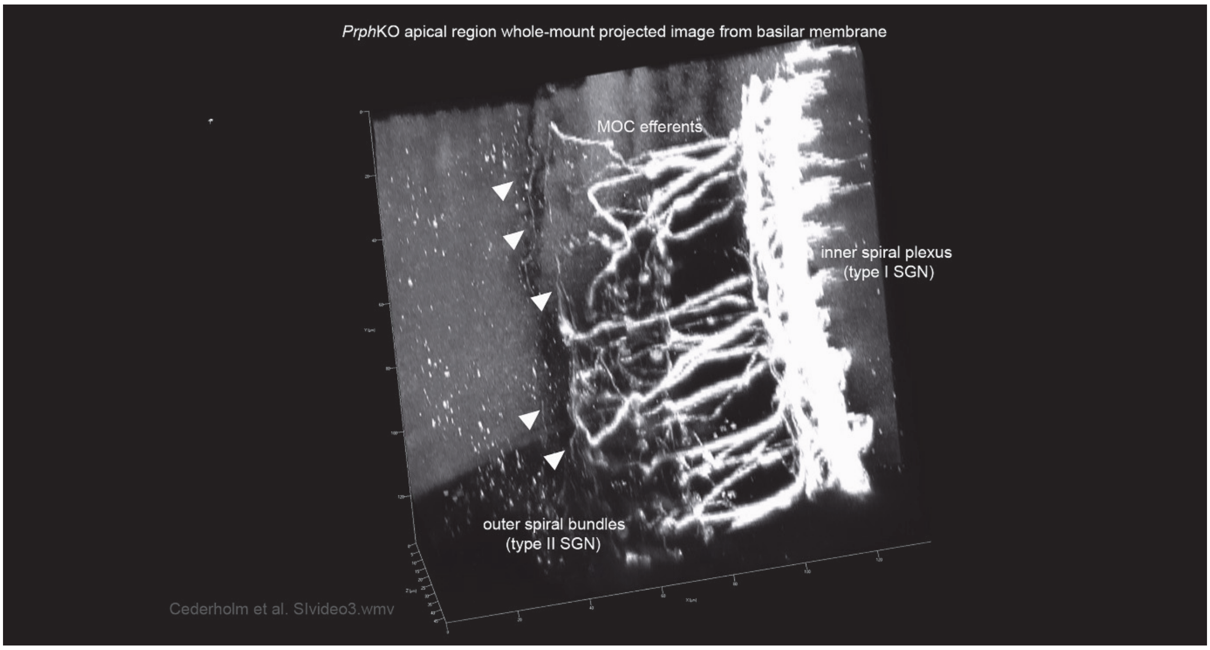

Supplementary Material 5: Video 3. β-III tubulin immunofluorescence of innervation of the organ of Corti in the apical region of the PrphKO mouse. *Whole-mount maximum intensity projection of a confocal image stack (48 µm) imaged from the basilar membrane surface from tissue batch-processed with the WT reference (SI Video 2). Note the reduced representation of Type II spiral ganglion afferents (type II SGN) in outer spiral bundles. Inner spiral plexus of type I spiral ganglion neurites (type I SGN) innervate the single row of inner hair cells. The medial olivocochlear (MOC) efferent fibres cross the tunnel of Corti to innervate the outer hair cells. This projection extends the visualisation of Figure 3B.* ***Filename: Cederholm et al. SIvideo3.wmv***

Cederholm *et al*. Frontiers in Neurology - Neuro-Otology. **Noise-induced hearing loss vulnerability in type III intermediate filament peripherin gene knockout mice**
Doi: 10.3389/fneur.2022.962227

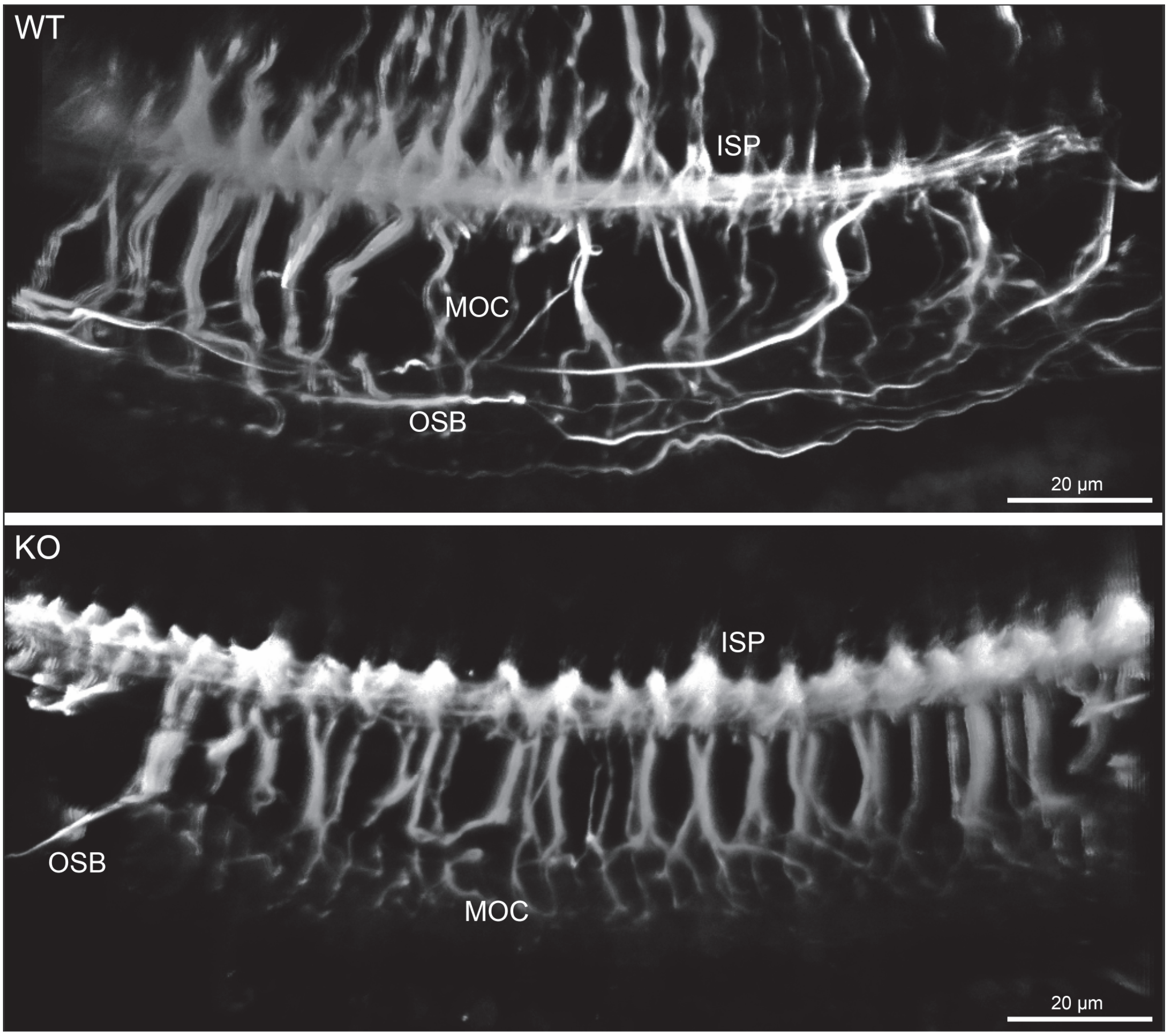

Supplementary Material 6: Figure 4 supplement 1. NF200 immunofluorescence labelling (maximum intensity projections) of batch-processed wholemounts from the apical region of wildtype (WT) and PrphKO organ of Corti*. Note the lack of outer spiral fibres within the outer spiral bundle (OSB; type II SGN afferent fibres) in the KO tissue, while medial olivocochlear (MOC) efferent projections to the outer hair cell region from the inner spiral plexus region (ISP) are equivalent. To optimally resolve the OSB fibre tracts, the tissue was imaged with the basilar membrane surface closest to the microscope objective, as the OSB lies beneath the MOC fibres.*

Cederholm *et al*. Frontiers in Neurology - Neuro-Otology. **Noise-induced hearing loss vulnerability in type III intermediate filament peripherin gene knockout mice**
Doi: 10.3389/fneur.2022.962227

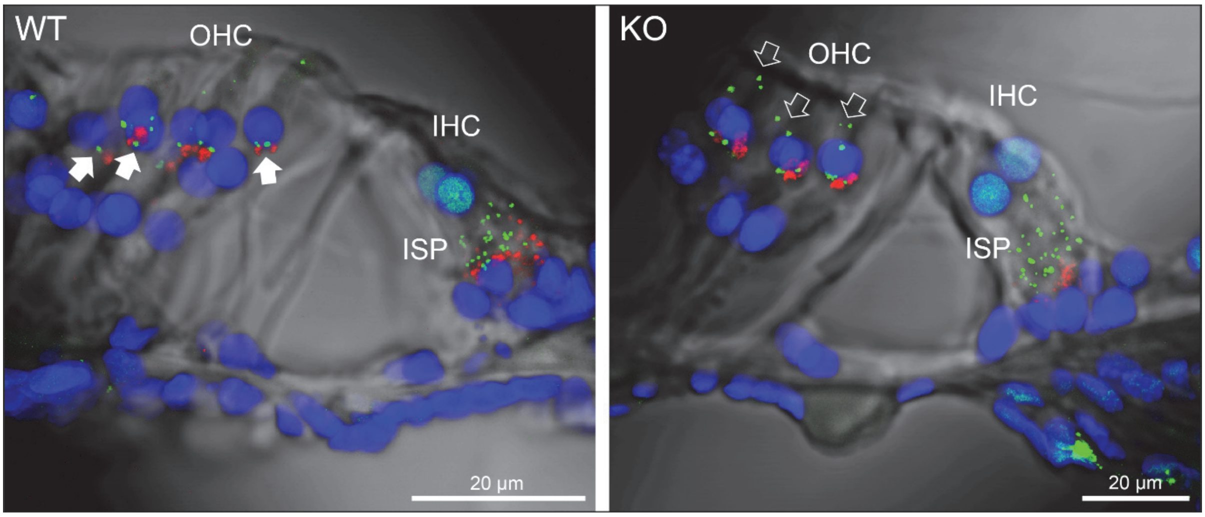

Supplementary Material 7: Figure 6 supplement 1. Evidence of disruption of the type II SGN afferent synapses with the outer hair cells (OHC) in the PrphKO cochlea*. CtBP2/RIBEYE immunofluorescence (green puncta) localizes pre-synaptic ribbon complexes at the base of OHCs and inner hair cells (IHC). While this labelling is consistent between WT and KO at the IHC synapses (inner spiral plexus region, ISP), there is pronounced apical migration of many of the synaptic ribbons away from the synaptic pole of the KO OHCs (compare filled arrows for WT and open arrows for KO). In contrast, the medial olivocochlear efferent synaptic boutons (VAChT immunofluorescence, red) are retained in the mid and medial aspects of the synaptic pole of the OHCs in the WT and KO cochlea. Nuclei labelled with DAPI. 50 µm cryosections.*

Doi: 10.3389/fneur.2022.962227

Supplementary Material 8:

Figure 6 - Source Data

Dataset for binned location of CtBP2 immunolabelled ribbons relative to outer hair cell nucleus equator in wildtype and *Prph*KO mouse cochlea confocal z stacks (1 – 3 per mouse)

Distribution of CtBP2 ribbons relative to centre of nucleus - WT mice

		WT#1	WT#2	WT#3	WT#4	WT#5		
basal	**12.5 to 15 µm**	0	0	0	0	0		
	**7.5 to 10**	0	0	0	0	0		
	**5 to 7.5**	0	0	0	0	0		
	**2.5 to; 5**	34	39	32	9	9	**#**	
basal	**0 to 2.5**	4	14	3	3	2	149	basal
apical	**0 to -2.5**	1	2	0	0	0	26	apical
	**-2.5 to -5**	6	2	0	1	3	175	total
	**-5 to -7.5**	3	0	3	0	0		
	**-7.5 to -10**	1	0	1	0	2		
	**-10 to -12.5**	0	0	0	0	1		
apical	**-12.5 to -15**	0	0	0	0	0		
	**n =**	49	57	39	13	17		

Distribution of CtBP2 ribbons relative to centre of nucleus - *Prph*KO mice

		KO#1	KO#2	KO#3	KO#4	KO#5	KO#6		
basal	**12.5 to 15 µm**	0	0	0	1	0	0		
	**7.5 to 10**	1	0	0	0	0	0		
	**5 to 7.5**	3	0	0	3	0	0		
	**2.5 to; 5**	26	14	33	6	10	1	**#**	
basal	**0 to 2.5**	6	6	1	10	2	2	125	basal
apical	**0 to -2.5**	2	1	1	2	2	2	89	apical
	**2.5 to -5**	8	3	8	7	5	1	214	Total
	**5 to -7.5**	3	2	7	3	0	0		
	**7.5 to -10**	6	1	9	2	2	0		
	**10 to -12.5**	4	0	1	4	0	0		
apical	**12.5 to -15**	1	0	0	2	0	0		
	**n =**	60	27	60	40	21	6		

Supplementary Material

Cederholm *et al*. Frontiers in Neurology - Neuro-Otology. **Noise-induced hearing loss vulnerability in type III intermediate filament peripherin gene knockout mice**

Doi: 10.3389/fneur.2022.962227

Supplementary Material 9:

Figure 9 - Source Data

Dataset for ABR threshold shift at 2 weeks post-noise
- 108 dB SPL

animal ID	genotype	frequency	threshold shift (dB)
1	*WT*	24 kHz	0
2	*WT*	24 kHz	15
3	*WT*	24 kHz	-5
4	*WT*	24 kHz	0
5	*WT*	24 kHz	-5
6	*WT*	24 kHz	0
7	*WT*	24 kHz	-10
8	*WT*	24 kHz	10
9	*WT*	24 kHz	0
1	*WT*	32 kHz	5
2	*WT*	32 kHz	25
3	*WT*	32 kHz	0
4	*WT*	32 kHz	7.5
5	*WT*	32 kHz	-5
6	*WT*	32 kHz	5
7	*WT*	32 kHz	-10
8	*WT*	32 kHz	30
9	*WT*	32 kHz	0
10	*PrphKO*	24 kHz	10
11	*PrphKO*	24 kHz	10
12	*PrphKO*	24 kHz	20
13	*PrphKO*	24 kHz	15
14	*PrphKO*	24 kHz	10
15	*PrphKO*	24 kHz	0
16	*PrphKO*	24 kHz	10
17	*PrphKO*	24 kHz	12.5
18	*PrphKO*	24 kHz	67.5*
10	*PrphKO*	32 kHz	15
11	*PrphKO*	32 kHz	25
12	*PrphKO*	32 kHz	30
13	*PrphKO*	32 kHz	10
14	*PrphKO*	32 kHz	10
15	*PrphKO*	32 kHz	0
16	*PrphKO*	32 kHz	15
17	*PrphKO*	32 kHz	17.5
18	*PrphKO*	32 kHz	52.5

* outlier identified by Grubb’s test, $\alpha = 0.05$

Doi: 10.3389/fneur.2022.962227

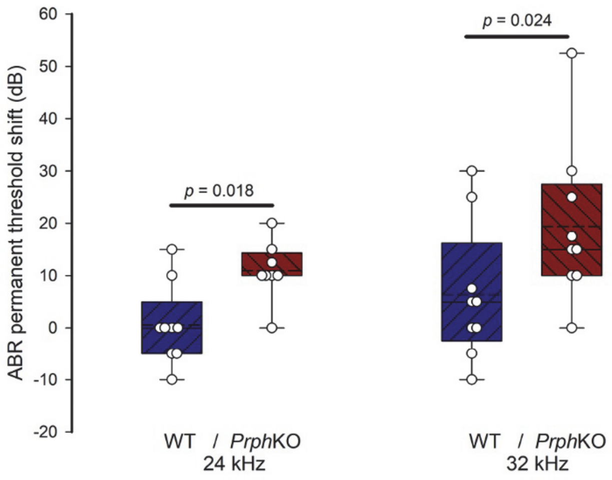

Supplementary Material 10: Figure 9 supplement 1. ABR threshold shifts indicative of increased vulnerability to noise-induced hearing loss in PrphKO mice*. Two weeks post – 108 dB SPL broadband noise for 1 hour. Boxplots show the 25% and 75 % boundaries and 95% limits along with overlaid data from 9 WT and 9 PrphKO mice; dashed line indicates mean, solid line indicates median. (Holm-Sidak post-hoc comparisons, repeated measures ANOVA;* Grubbs' test (GraphPad) excluded one data point as an outlier for *Prph*KO 24 kHz)). *See also Supplementary Material 10.*

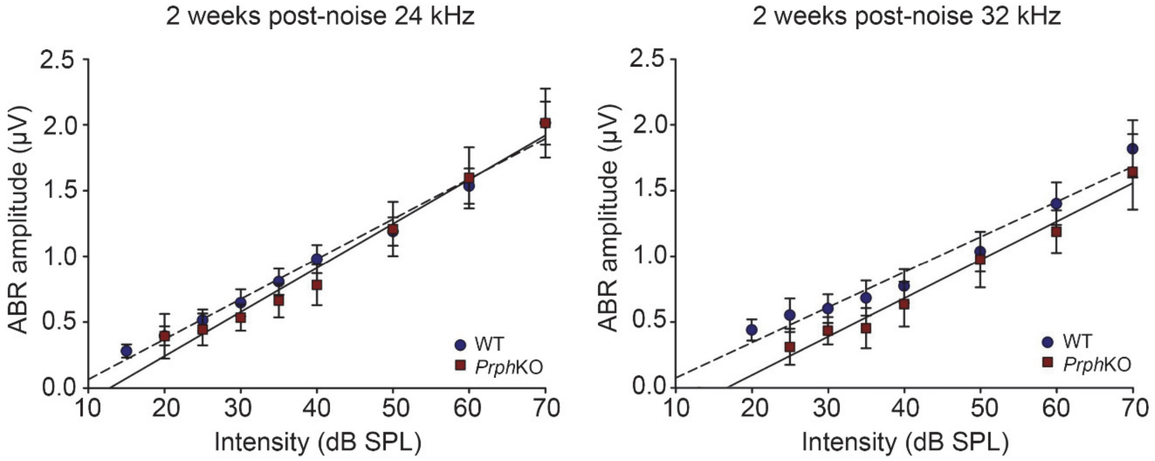

Supplementary Material 11: Figure 9 supplement 2. Suprathreshold ABR wave N1 – P2 amplitudes reflect neural recruitment with increasing sound intensity two weeks after 108 dB SPL white noise exposure in WT mice, and the PrphKO mice with significant hearing loss*. The slopes of the linear regression best fits to these input / output functions at 24 kHz and 32 kHz were not significantly different across genotype (n = 8 WT; n = 7 PrphKO – solid lines). Average slopes & R^2 values: 24 kHz: WT 0.030 µV / dB SPL & 0.988; KO 0.033 µV / dB SPL & 0.975; 32 kHz: WT 0.026 µV / dB SPL & 0.962; KO 0.029 µV / dB SPL & 0.98).*

Doi: 10.3389/fneur.2022.962227

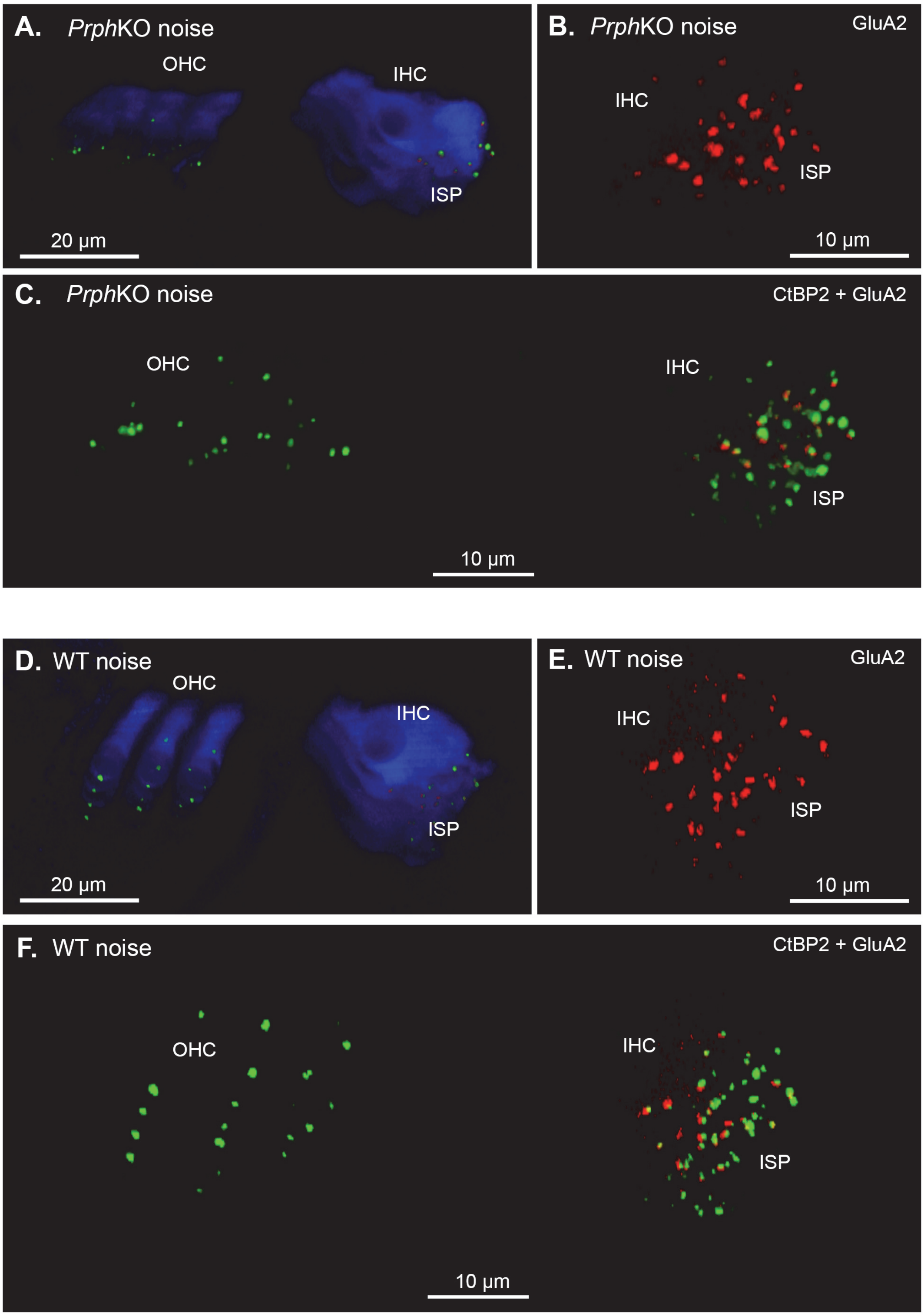

Supplementary Material 12: Figure 9 supplement 3. Immunofluorescence imaging of noise-exposed cochleae. *Hair cell integrity and immunolabelling of synaptic markers were unremarkable in both PrphKO and wildtype (WT) cochleae two weeks post-noise (108 dB SPL, 4 – 32 kHz, 1 hour). 50 µm cochlear cryosections, lower-basal region; 3D rendered confocal image stacks. A. Myosin 7A (blue; detected using an Alexa647 goat anti-rabbit*

Doi: 10.3389/fneur.2022.962227

secondary antibody) immunolabelling delineates outer hair cell (OHC) and inner hair cell (IHC) integrity; punctate CtBP2/RIBEYE labelling (green; Alexa488 goat anti-mouse IgG1 secondary antibody) localizes pre-synaptic ribbons, which in the inner spiral plexus region (ISP) at IHC is confluent with post-synaptic AMPA type 2 glutamate receptor immunolocalization (GluA2; red, Alexa594 goat anti-mouse IgG2A). B. Detail of the GluA2 immunolocalization from A. C. Detail of the CtPB2 and GluA2 colocalization at the IHC; disruption in basal pre-synaptic ribbon localization on the OHC, which is a PrphKO phenotype characteristic, was maintained. (from A). D. WT lower-basal region illustrating hair cell and synapse integrity (Myosin7A + CtBP2 +GluA2 immunolabelling). E. Detail of post-synaptic GluA2 localization at IHC type I afferent synapses. F. Co-immunolocalization of CtBP2 pre-synaptic ribbons and GluA2 post-synaptic afferent synapses at the IHC.
